# Integrated Pan-Cancer Analysis and Functional Characterization of ATPSCKMT in Hepatocellular Carcinoma

**DOI:** 10.3390/molecules31213887

**Published:** 2026-10-28

**Authors:** Soon Hyung Kwon, Ji Eon Ha, Sae-Ock Oh, Sang-Mo Kwon

**Affiliations:** 1 Convergence Stem Cell Research Center, Pusan National University, Yangsan 50612, Republic of Korea; shk705@naver.com (S.H.K.); dzelha_@naver.com (J.E.H.); 2 Laboratory for Vascular Medicine and Stem Cell Biology, Department of Physiology, School of Medicine, Pusan National University, Yangsan 50612, Republic of Korea; 3 Department of Anatomy, School of Medicine, Pusan National University, 49 Busandaehak-ro, Yangsan 50612, Republic of Korea; 4 Research Center for Molecular Control for Cancer Cell Diversity, Pusan National University, Yangsan 50612, Republic of Korea; 5 Research Institute of Convergence Biomedical Science and Technology, Pusan National University Yangsan Hospital, Yangsan 50612, Republic of Korea

**Keywords:** ATPSCKMT, hepatocellular carcinoma, methyltransferase, METTL family, seven-β-strand lysine methyltransferase, cancer dependency, CCT5, pan-cancer analysis

## Abstract

Hepatocellular carcinoma (HCC) remains a major cause of cancer mortality, highlighting the need to identify functionally relevant molecular determinants. We systematically evaluated 38 genes classified as members of the methyltransferase-like (METTL) family and/or seven-β-strand lysine methyltransferases using pan-cancer genomic, transcriptomic, survival, and dependency datasets, followed by functional assessment in HCC cell lines. Integrated analysis prioritized ATP synthase c-subunit lysine methyltransferase (ATPSCKMT) for investigation in liver hepatocellular carcinoma (LIHC), where elevated expression was associated with adverse prognosis. DEMETER2 analysis also identified widespread RNAi dependency across cancer types. siRNA-mediated ATPSCKMT knockdown in JHH7 and HEP3B cells reduced CCK-8 signals and EdU incorporation. Network and co-expression analyses identified CCT5 as a representative ATPSCKMT-associated candidate, with reproducible positive correlations across independent HCC cohorts. In TCGA-LIHC, the ATPSCKMT–CCT5 correlation persisted after adjustment for shared local copy-number variation (partial Spearman ρ = 0.522). Single-cell analysis of primary HCC malignant hepatocytes further revealed patient-level co-detection, although these findings may be influenced by differences in transcript detection. Overall, our findings support further investigation of ATPSCKMT in HCC proliferation-associated phenotypes and identify CCT5 as a reproducible co-expression candidate.

## 1. Introduction

Primary liver cancer is a major malignancy with a high global mortality burden, and hepatocellular carcinoma (HCC) accounts for the majority of primary liver cancers [1,2]. HCC is characterized by diverse etiologies, pronounced molecular heterogeneity, and substantial intratumoral variation, while post-treatment recurrence and therapeutic resistance remain major obstacles to long-term survival [2,3,4,5,6]. Although large-scale genomic and transcriptomic studies have revealed the molecular diversity and recurrent genomic alterations of HCC [7,8], further efforts are required to identify and functionally validate molecular determinants that contribute to cancer-cell proliferation and survival. In particular, systematic comparisons of genomic alterations, expression patterns, and functional requirements across cancer types, without prespecifying a particular gene or pathway, may facilitate the objective identification of new candidates for investigation in HCC.

Methyltransferases generally use S-adenosyl-L-methionine (SAM) as a methyl donor to transfer methyl groups to RNA, proteins, and other cellular substrates [9,10]. Protein lysine methylation is a post-translational modification that occurs not only on histones but also on diverse non-histone proteins, including transcription factors, molecular chaperones, translation factors, and mitochondrial proteins [10,11,12,13]. This modification can affect a broad range of cellular functions by altering protein structure, enzymatic activity, stability, and molecular interactions [10,13,14]. Protein lysine methyltransferases (KMTs) are classified primarily into SET-domain and seven-β-strand (7BS) methyltransferases according to the architecture of their catalytic domains [9,11]. Although 7BS methyltransferases share a SAM-dependent catalytic architecture, differences in their substrate-recognition elements confer diverse substrate specificities [9,11]. Many methyltransferases bearing the methyltransferase-like (METTL) designation adopt a 7BS architecture, whereas some structurally related 7BS lysine methyltransferases have distinct gene names based on their target substrates [9,12]. Consequently, restricting an analysis to genes bearing the METTL designation may exclude structurally and functionally related methyltransferases. We therefore assembled an expanded METTL/7BS KMT analysis set comprising genes classified as members of the METTL family and/or as seven-β-strand lysine methyltransferases. The 38 genes were categorized into three groups: METTL family only (n = 24), METTL family + 7BS KMT (n = 9), and 7BS KMT only (n = 5) (Table 1). This classification was used as an analytical framework for the present study and does not imply that all included genes constitute a single enzyme family with a common substrate [12].

Functionally characterized METTL methyltransferases participate in diverse biological processes, including RNA metabolism, translation, protein homeostasis, mitochondrial function, and cell proliferation [9,12,15]. Several members, including METTL3 and METTL1, are dysregulated in HCC and other cancers and have been implicated in cancer-cell proliferation, tumor progression, and therapeutic responses [16,17,18,19,20]. Although a previous pan-cancer study integrated genomic, transcriptomic, proteomic, clinical, and loss-of-function features of METTL-related genes [21], structurally defined non-METTL 7BS lysine methyltransferases have been less systematically incorporated into such comparative cancer frameworks. In particular, genomic alterations, tumor-versus-normal expression differences, patient outcomes, and cancer-cell dependencies remain incompletely compared across an expanded analysis set that jointly considers METTL-named genes and structurally related non-METTL 7BS KMTs. A pan-cancer assessment of this expanded gene set is therefore warranted to identify candidates supported by convergent evidence from multiple analytical domains.

ATP synthase c subunit lysine N-methyltransferase (ATPSCKMT), formerly known as FAM173B, is a mitochondrial 7BS lysine methyltransferase [22,23,24]. ATPSCKMT trimethylates Lys-43 of the mitochondrial ATP synthase c-subunit [22,23]. Loss of Lys-43 methylation following ATPSCKMT deficiency has been associated with aberrant incorporation of the c-subunit into the ATP synthase complex, reduced ATP-generating capacity, and impaired mitochondrial respiration [22,23]. Although biochemical studies have established the substrate and mitochondrial function of ATPSCKMT, its genomic alterations and expression patterns across cancers, as well as their relationships with cancer-cell dependency and patient outcomes, have not been systematically evaluated. In particular, the expression, proliferation-related function, and molecular features of ATPSCKMT in HCC remain poorly defined.

In this study, we systematically analyzed genomic alterations, tumor-versus-normal differential expression, patient outcomes, and RNA interference (RNAi) dependency across the expanded METTL/7BS KMT analysis set at the pan-cancer level. On the basis of convergent evidence from these analyses, ATPSCKMT was selected as a representative candidate for further functional investigation. Expression, prognostic, and RNAi dependency evidence for ATPSCKMT was then integrated across cancer types to prioritize HCC for subsequent study. The reproducibility of ATPSCKMT expression and its proliferation-related function in HCC were evaluated using independent cohorts and cell-based experiments, while bulk and single-cell transcriptomic analyses were used to characterize proliferation-associated molecular features and candidate genes linked to ATPSCKMT. This study therefore aimed to provide a systematic pan-cancer characterization of the expanded METTL/7BS KMT analysis set and to evaluate the functional relevance and associated molecular features of ATPSCKMT in HCC.

## 2. Results

### 2.1. Recurrent Genomic Alterations of METTL and 7BS KMT Genes Are Associated with Expression and Patient Survival Across Cancers

**Figure 1:**
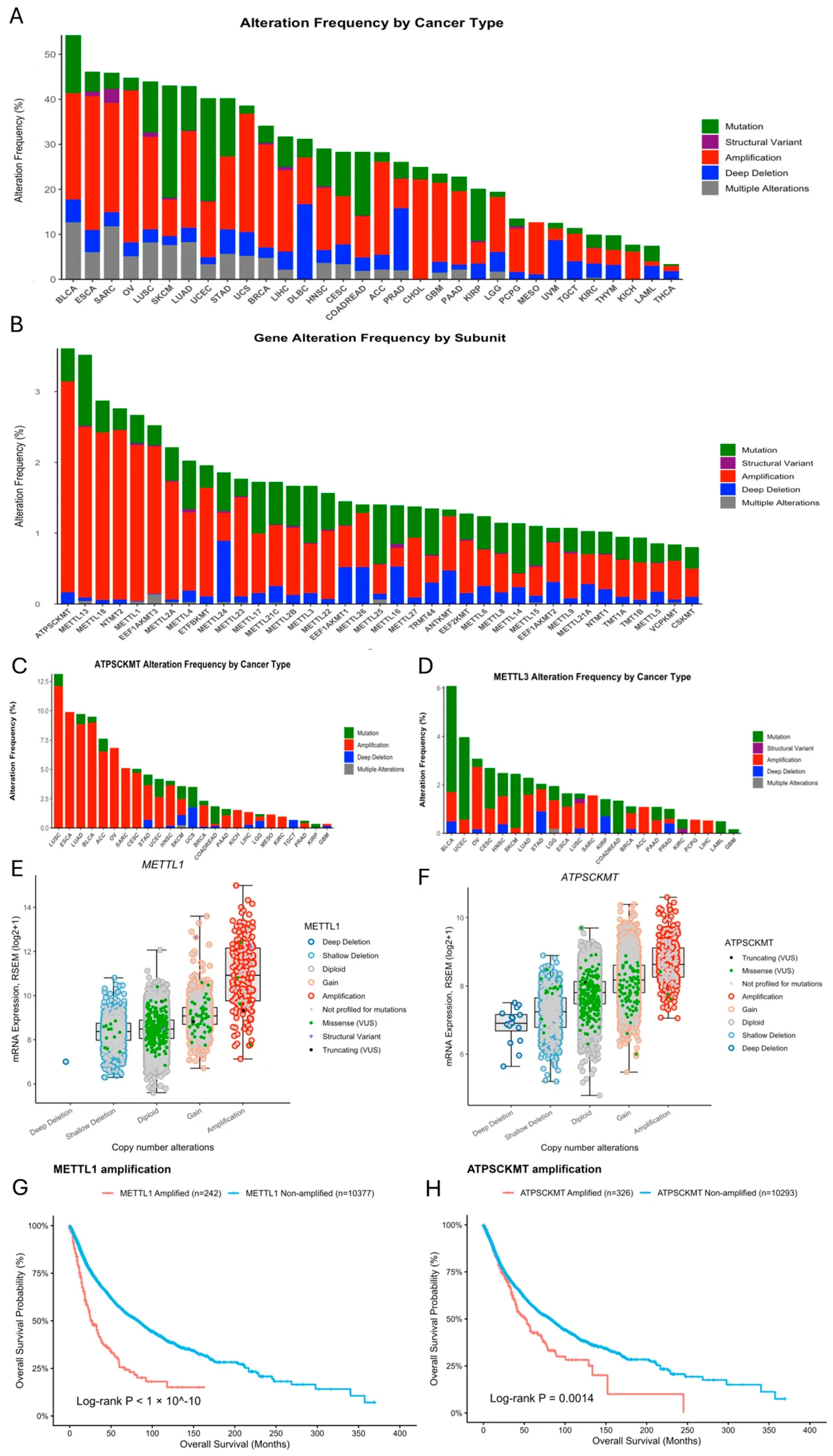
Pan-cancer genomic alteration landscape and molecular characterization of METTL and 7BS lysine methyltransferase genes. (**A**) Frequency and composition of genomic alterations in METTL and 7BS lysine methyltransferase (7BS KMT) genes across TCGA cancer types. Stacked bars indicate mutations, structural variants, amplifications, deep deletions, and multiple alterations. (**B**) Gene-level genomic alteration frequencies of individual METTL/7BS KMT genes across the TCGA pan-cancer cohort. Genes are ordered according to overall alteration frequency, and colors indicate the corresponding alteration types. (**C**,**D**) Cancer type-specific genomic alteration frequencies of ATPSCKMT (**C**) and METTL3 (**D**), representing the highest-ranked genes in the amplification and mutation categories, respectively. (**E**,**F**) mRNA expression levels of METTL1 (**E**) and ATPSCKMT (**F**), the two genes with the highest amplification frequencies, according to copy-number status. Samples were classified as deep deletion, shallow deletion, diploid, gain, or amplification. Each point represents an individual tumor sample. (**G**) Overall survival of patients with METTL1 amplification compared with patients without METTL1 amplification. (**H**) Overall survival of patients with ATPSCKMT amplification compared with patients without ATPSCKMT amplification.

We assembled an expanded analysis set of 38 genes classified as METTL family only (n = 24), METTL family + 7BS KMT (n = 9), or 7BS KMT only (n = 5) (Table 1). Analysis of TCGA PanCancer Atlas data obtained from cBioPortal [25,26] revealed that mutations, structural variants, amplifications, deep deletions, and multiple alterations were widely distributed across cancer types, while the overall alteration frequency and predominant alteration type differed among cancers (Figure 1A). At the individual-gene level, amplification and mutation were the major genomic alteration types observed in the METTL/7BS KMT gene set (Figure 1B). Analysis of cancer type-specific distributions for the leading genes in each alteration category showed that ATPSCKMT, which had the highest amplification frequency, was altered predominantly through copy-number amplification across multiple cancer types (Figure 1C). In contrast, METTL3, which had the highest mutation frequency, exhibited mutation-dominant alteration patterns across diverse cancer types (Figure 1D).

We next compared the mRNA expression of METTL1 and ATPSCKMT, the genes with the highest amplification frequencies, according to copy-number status. For both genes, mRNA expression progressively increased with copy number and was highest in the amplification group (Figure 1E,F). Kaplan–Meier analyses were then performed to evaluate the clinical associations of copy-number amplification. Patients with METTL1 amplification (n = 242) had significantly shorter overall survival than those without amplification (n = 10,377; log-rank *p* < 1 × 10^−10^; Figure 1G). Similarly, patients with ATPSCKMT amplification (n = 326) had significantly shorter overall survival than those without amplification (n = 10,293; log-rank *p* = 0.0014; Figure 1H). Thus, copy-number amplification of METTL1 and ATPSCKMT was associated with increased mRNA expression and adverse patient survival.

For METTL3 and METTL4, which showed high mutation frequencies, we additionally examined the distribution of mutations across their protein sequences and their associations with patient survival. Mutations in both genes were distributed throughout the protein sequences, with recurrent R508G/C/H alterations within the MT-A70 domain of METTL3 and recurrent W449*/C/R alterations in METTL4 (Supplementary Figure S1A,B). Kaplan–Meier analysis showed that the METTL3-mutated group (n = 88) had a significantly different overall-survival distribution from the non-mutated group (n = 10,209; log-rank *p* = 0.0321; Supplementary Figure S1C). A significant survival difference was also observed between the METTL4-mutated group (n = 76) and the non-mutated group (n = 10,221; log-rank *p* = 0.0383; Supplementary Figure S1D). During early follow-up, both mutation groups showed higher survival probabilities than their corresponding non-mutated groups, indicating survival patterns distinct from those associated with amplification. Collectively, these findings demonstrate that genomic alterations of METTL/7BS KMT genes occur across multiple cancers and that their clinical associations differ according to the affected gene and alteration type.

### 2.2. METTL/7BS KMT Genes Exhibit Widespread Dysregulation with Predominant Upregulation Across Cancer Types

**Figure 2:**
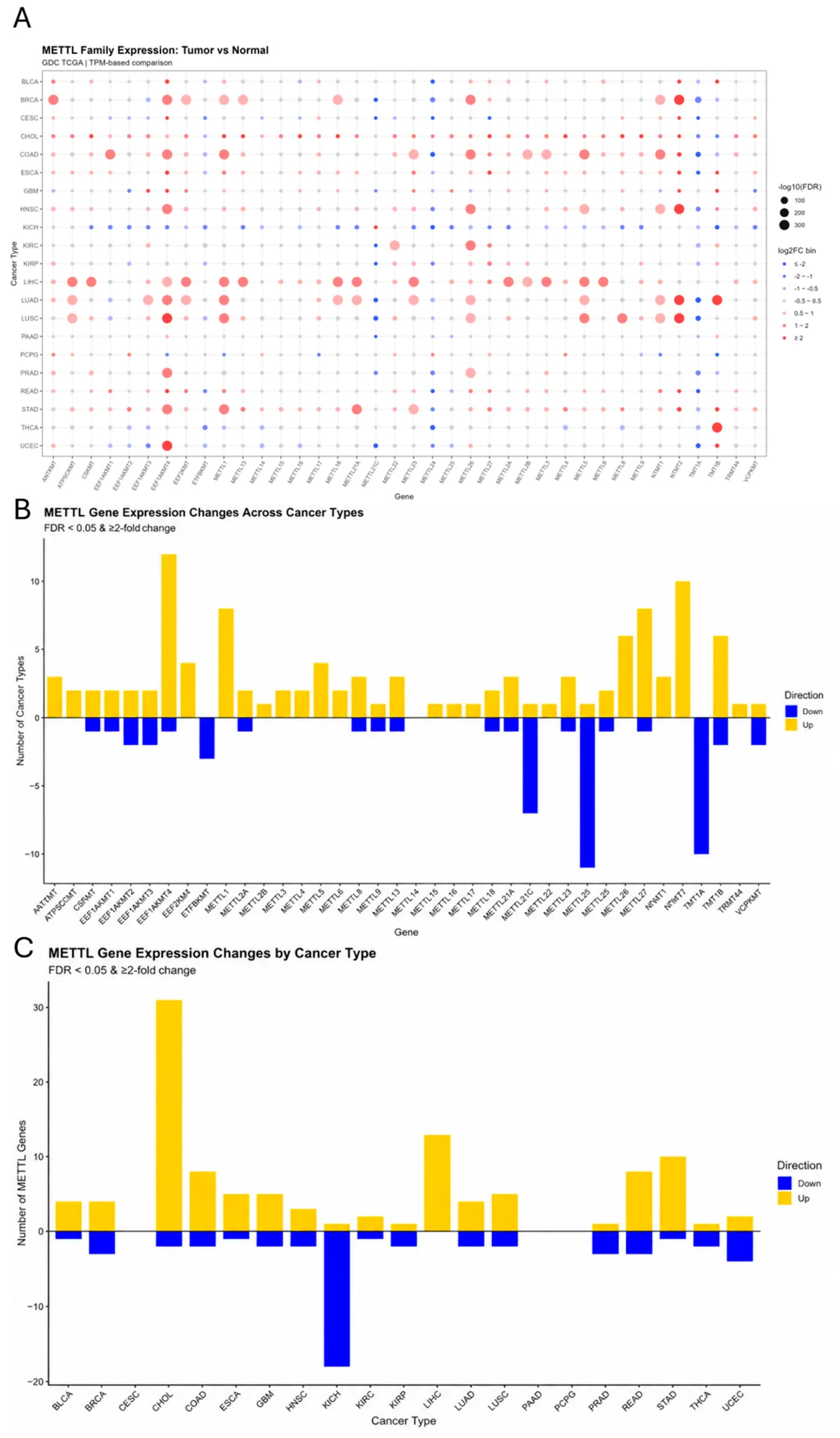
Pan-cancer differential expression landscape of METTL and 7BS lysine methyltransferase genes. (**A**) Differential expression of METTL/7BS KMT genes between tumor and normal tissues across 21 GDC–TCGA cancer types. Dot color indicates the direction and magnitude of the log2 fold change, with red and blue representing increased and decreased expression, respectively. Dot size is proportional to −log10(FDR), with larger dots indicating greater statistical significance. (**B**) Number of cancer types in which each METTL/7BS KMT gene was significantly upregulated or downregulated. (**C**) Number of significantly upregulated or downregulated METTL/7BS KMT genes within each cancer type. In (**B**,**C**), significant differential expression was defined as FDR < 0.05 and |log2 fold change| ≥ 1. Yellow and blue bars indicate upregulation and downregulation, respectively.

To evaluate cancer-associated expression changes in the METTL/7BS KMT gene set, we compared mRNA expression between tumor and normal tissues across 21 TCGA cancer types [27]. Numerous METTL/7BS KMT genes showed significant expression changes in multiple cancers, although the direction and magnitude of these changes differed according to the gene and cancer type (Figure 2A). When the numbers of cancer types showing significant expression changes were compared for each gene, most genes were upregulated in more cancer types than they were downregulated (Figure 2B). EEF1AKMT4 showed the broadest upregulation and was significantly increased in 12 cancer types, whereas METTL24 showed the broadest downregulation and was significantly decreased in 11 cancer types.

We next examined the number of significantly altered genes within each cancer type. CHOL showed the most extensive upregulation, with 31 upregulated and two downregulated genes (Figure 2C). In liver hepatocellular carcinoma (LIHC), all 13 genes meeting the predefined criteria were upregulated, whereas STAD had 10 upregulated and one downregulated gene. In contrast, KICH displayed a distinct expression pattern, with 18 downregulated genes and only one upregulated gene. Collectively, dysregulation of the METTL/7BS KMT gene set was not restricted to a specific gene or cancer type, and upregulation in tumor tissues predominated overall. These results identify transcriptional dysregulation of METTL/7BS KMT genes as a recurrent cancer-associated molecular feature across cancer types.

### 2.3. METTL/7BS KMT Gene Expression Is Recurrently Associated with Patient Prognosis Across Cancer Types

**Figure 3:**
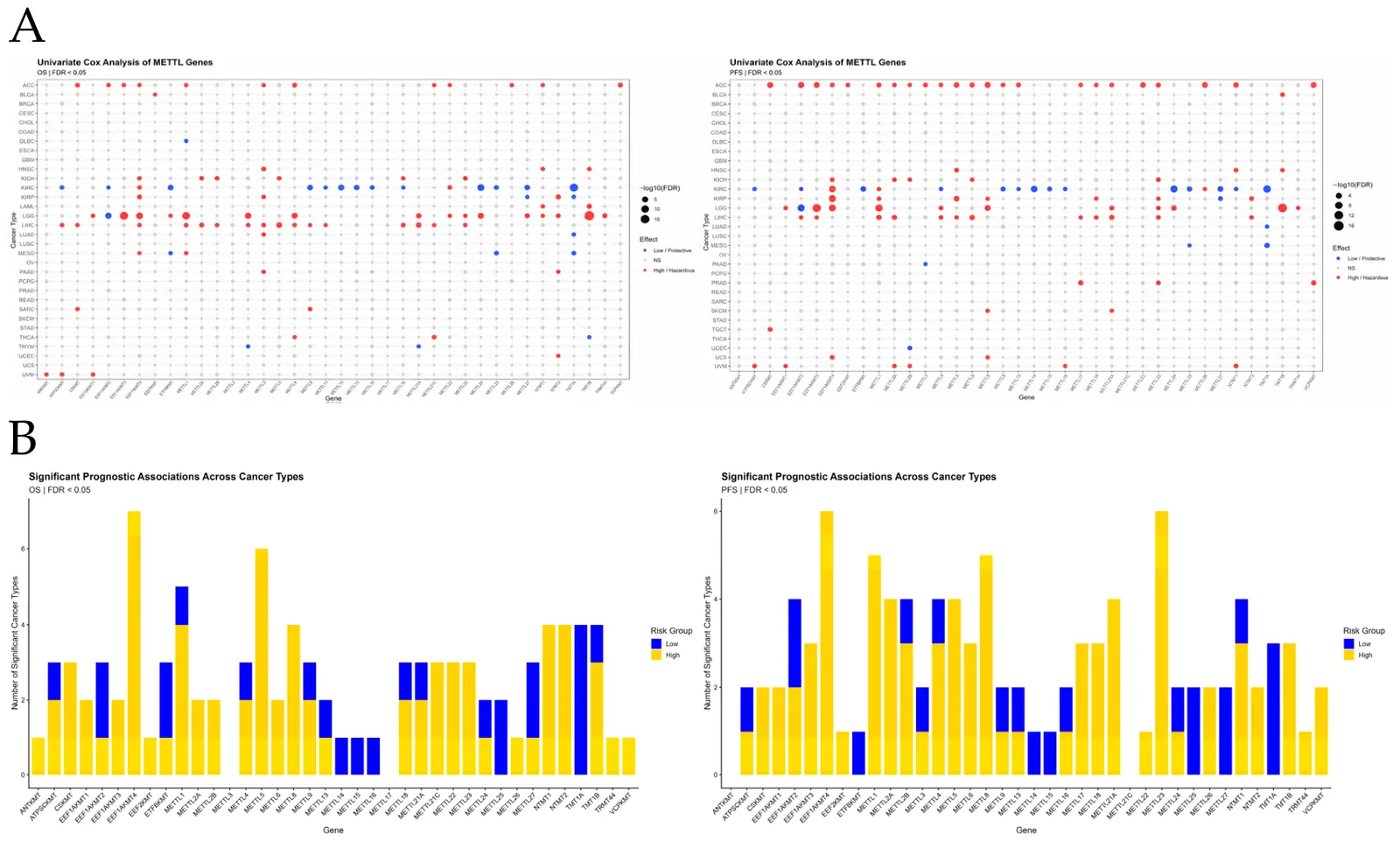

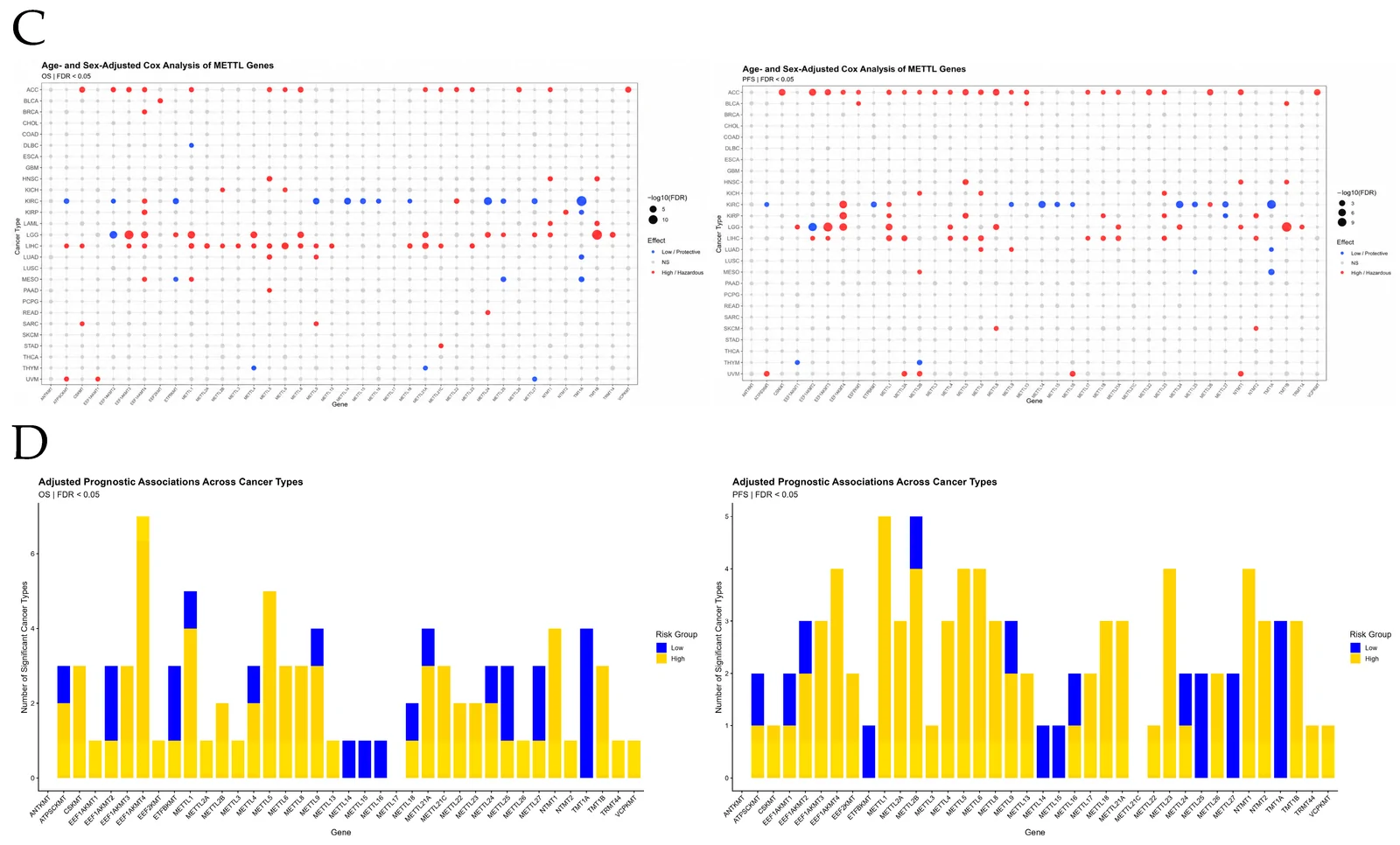
Pan-cancer prognostic associations of METTL and 7BS lysine methyltransferase gene expression. (**A**) Univariate Cox proportional hazards regression analyses of METTL/7BS KMT gene expression for overall survival (OS; (**left**)) and progression-free interval (PFI; (**right**)) across TCGA cancer types. Red and blue dots indicate high-risk and low-risk associations, respectively, whereas gray dots indicate non-significant associations. In (**B**,**D**), yellow and blue bars indicate high-risk and low-risk associations, respectively. Dot size represents −log10(raw *p* value), whereas the dot color in (**A**,**C**) and the bar color in (**B**,**D**) denote statistical significance defined as an FDR-adjusted *p* value < 0.05. (**B**) Number of cancer types showing significant high- or low-risk associations for each gene in the univariate OS (**left**) and PFI (**right**) analyses. (**C**) Cox proportional hazards regression analyses of OS (**left**) and PFI (**right**) after adjustment for age and sex. Dot color and size are defined as in (**A**). (**D**) Number of cancer types showing significant high- or low-risk associations for each gene in the age- and sex-adjusted OS (**left**) and PFI (**right**) analyses.

To evaluate the relationship between METTL/7BS KMT gene expression and patient outcomes, Cox proportional hazards regression analyses were performed across TCGA cancer types [28]. In univariate analyses, the expression of multiple METTL/7BS KMT genes was significantly associated with overall survival (OS) and progression-free interval (PFI) in several cancers (Figure 3A). Although the direction of the prognostic association differed according to the gene and cancer type, high expression was more frequently associated with adverse outcomes than with favorable outcomes. At the gene level, significant prognostic associations were detected in up to seven cancer types for OS and up to six cancer types for PFI (Figure 3B). For many genes, adverse associations were more frequent than favorable associations, indicating that high expression of METTL/7BS KMT genes was recurrently associated with unfavorable outcomes across cancers. After adjustment for age and sex, significant associations between METTL/7BS KMT gene expression and OS or PFI remained detectable in multiple cancer types (Figure 3C). Following adjustment, individual genes were prognostically significant in up to seven cancer types for OS and up to five cancer types for PFI, and the overall predominance of adverse associations was maintained (Figure 3D). Collectively, METTL/7BS KMT gene expression was recurrently associated with patient outcomes across cancer types, and these associations were broadly retained after adjustment for age and sex. These findings suggest that dysregulated expression of the METTL/7BS KMT gene set represents a widespread prognostic feature in cancer.

### 2.4. Dependency Profiling of METTL/7BS KMT Genes Identifies ATPSCKMT as a Broadly Knockdown-Sensitive Gene Across Cancer Cell-Line Groups

**Figure 4:**
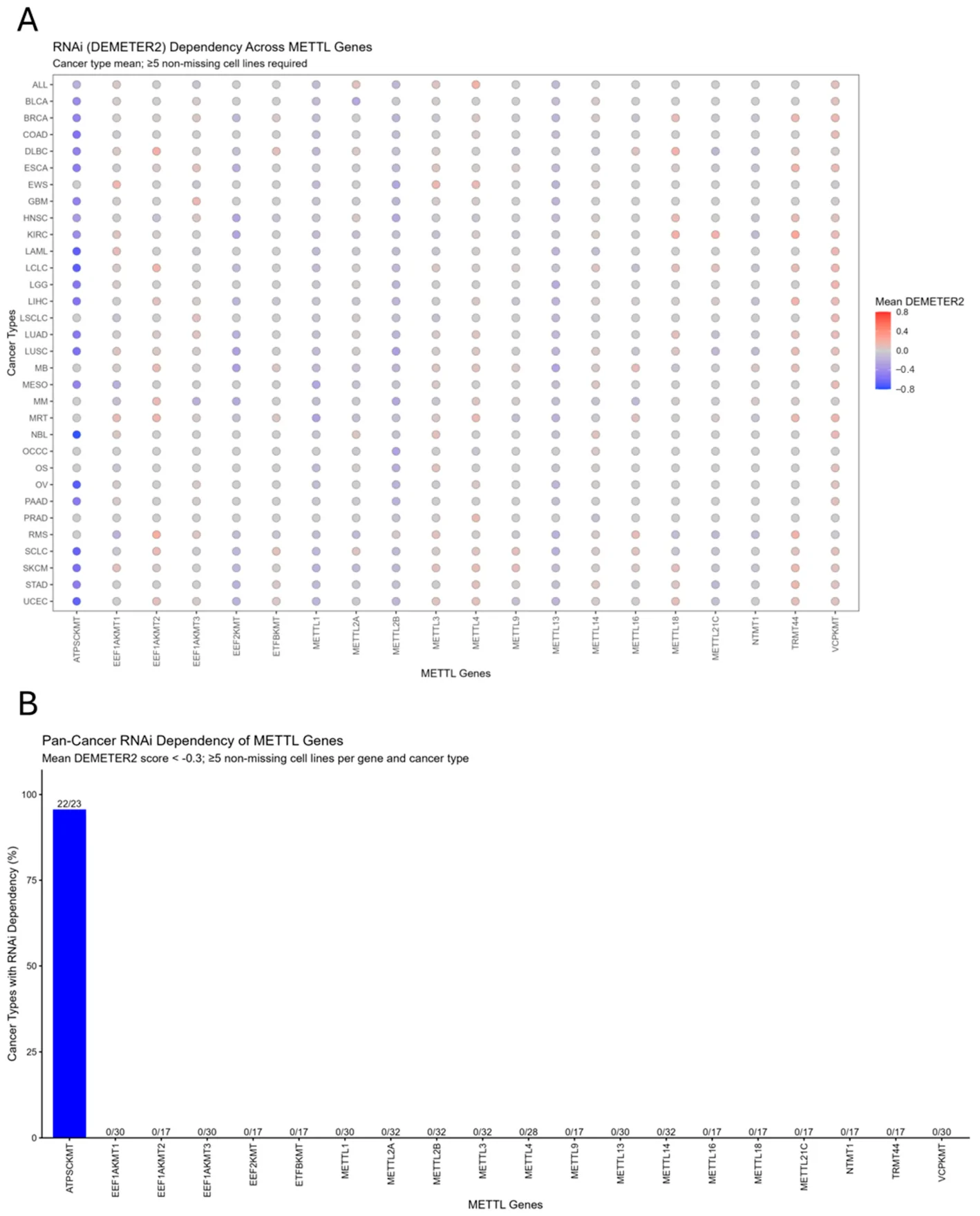
Pan-cancer RNAi dependency landscape of METTL and 7BS lysine methyltransferase genes. (**A**) Mean DEMETER2 RNAi dependency scores of METTL/7BS KMT genes across cancer cell-line groups. Gene–cancer type combinations with at least five cell lines containing non-missing DEMETER2 values were included. Dot color represents the mean DEMETER2 score on a continuous scale, with more negative values indicating stronger dependency. Gene–cancer type combinations with fewer than five non-missing observations were treated as unavailable. (**B**) Proportion of evaluable cancer types meeting the predefined RNAi dependency criterion for each METTL/7BS KMT gene. RNAi dependency was defined as a mean DEMETER2 score < −0.3. Numbers above the bars indicate the number of cancer types meeting the criterion relative to the total number of evaluable cancer types for each gene.

To determine whether METTL/7BS KMT genes are functionally required for cancer-cell survival and proliferation, we performed a pan-cancer RNAi dependency analysis using DepMap DEMETER2 data [29]. For the 20 METTL/7BS KMT genes represented in the DEMETER2 dataset, mean dependency scores were calculated for each cancer type-gene combination containing at least five cell lines with non-missing data. ATPSCKMT consistently showed negative DEMETER2 scores in most evaluable cancer types and exhibited a generally stronger RNAi dependency pattern than the other METTL/7BS KMT genes (Figure 4A). Using a mean DEMETER2 score of less than −0.3 as the RNAi dependency criterion, ATPSCKMT met this threshold in 22 of 23 evaluable cancer types (95.7%; Figure 4B). In contrast, none of the remaining METTL/7BS KMT genes met the same criterion in any evaluable cancer type. Thus, among the evaluable METTL/7BS KMT genes, ATPSCKMT exhibited the broadest and most consistent RNAi dependency across cancers. These findings suggest that reduced ATPSCKMT expression is associated with impaired fitness in diverse cancer-cell groups and provide a rationale for prioritizing ATPSCKMT for subsequent cancer type-specific analyses and functional validation. To characterize the dependency behavior of ATPSCKMT across perturbation modalities, we performed a parallel analysis of CRISPR–Cas9 gene-effect scores processed with the Chronos model. Of the 38 genes in the analysis set, all 38 were evaluable in the Chronos dataset. The two resources differ in perturbation mechanism, cell-line coverage, and model classification and yielded different numbers of evaluable cancer-type categories (23 for DEMETER2 versus 45 for Chronos); the corresponding proportions are therefore not directly comparable and were interpreted within each platform. Applying the same descriptive threshold of a mean gene-effect score < −0.3, ATPSCKMT did not meet the criterion in any of the 45 evaluable cancer-type groups in the Chronos dataset, whereas 12 other genes met this threshold in at least one group (Supplementary Figure S2). These findings indicate a substantial discrepancy between the RNAi- and CRISPR-based dependency profiles of ATPSCKMT. Although previous biochemical studies demonstrated that ATPSCKMT-mediated Lys-43 methylation optimizes ATP synthase function [23], the present analyses do not establish the cause of this discrepancy. Cancer-type prioritization was based on the DEMETER2 analysis, and the Chronos results are reported separately as complementary evidence.

### 2.5. Integrated Pan-Cancer Evidence Prioritizes LIHC for Further Investigation of ATPSCKMT

Based on the convergent genomic, prognostic, and RNAi dependency findings, ATPSCKMT was selected for cancer type-specific prioritization. Although other members of the analysis set, including METTL1 and METTL3, have been extensively investigated in HCC [16,17,18,19,20], the cancer-associated relevance of ATPSCKMT remains comparatively understudied despite its established mitochondrial enzymatic function. We therefore integrated expression, prognosis, and RNAi dependency evidence to identify a cancer type for further investigation.

**Figure 5:**
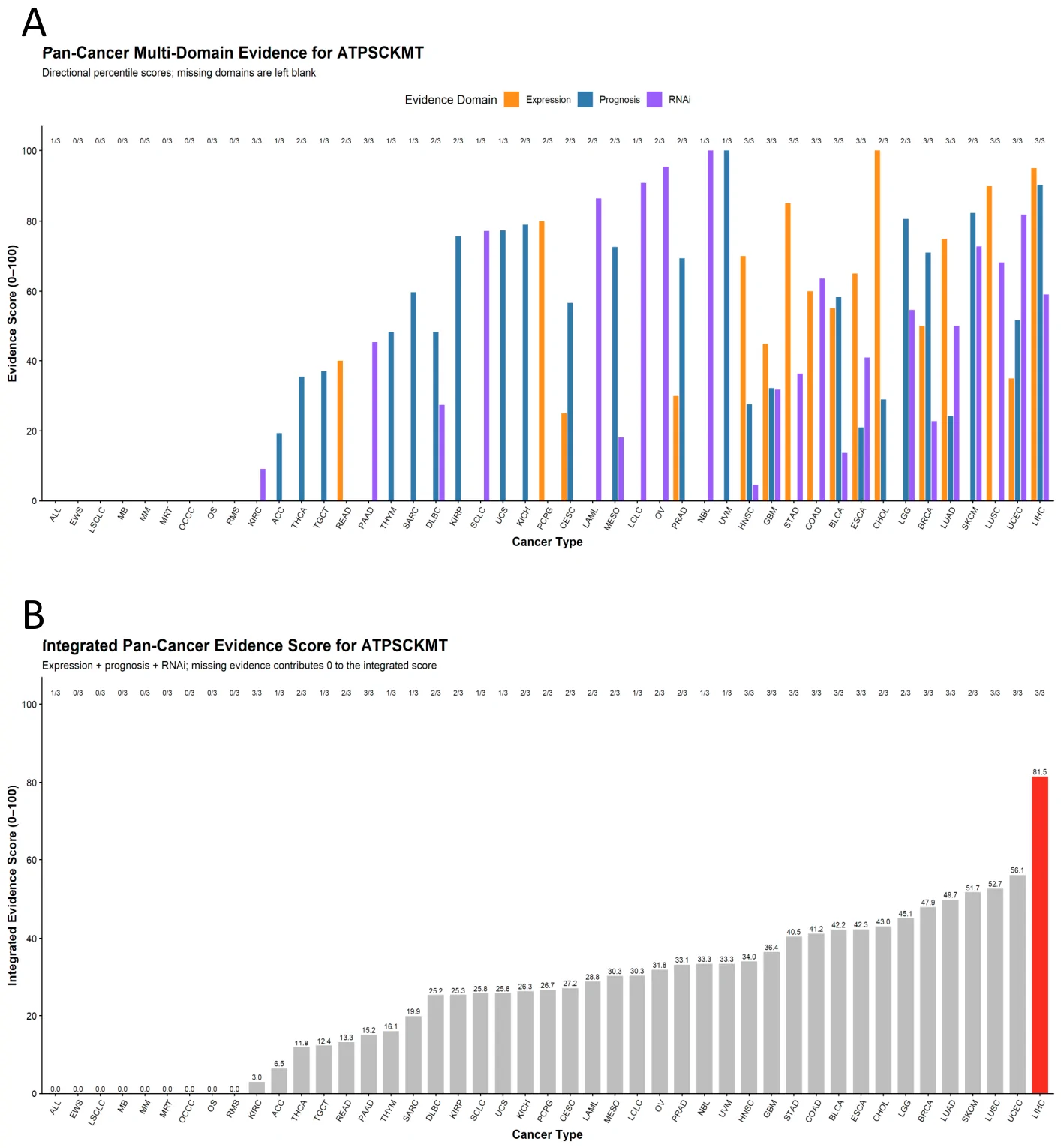
Integrated pan-cancer prioritization of ATPSCKMT based on expression, prognosis, and RNAi dependency. (**A**) Cancer type-specific evidence scores for ATPSCKMT independently derived from differential expression, prognostic association, and RNAi dependency analyses. Each evidence domain was transformed into a directional percentile score ranging from 0 to 100, with higher scores representing stronger tumor-associated upregulation, adverse prognostic association, or RNAi dependency. Opposite-direction effects were assigned a score of zero, whereas unavailable domains were left blank. Fractions above each cancer type indicate the number of available evidence domains out of three and represent data completeness rather than the number of positive evidence domains. (**B**) Integrated evidence scores calculated as the equally weighted arithmetic mean of the expression, prognosis, and RNAi dependency scores. Unavailable domains were assigned a score of zero so that the integrated score incorporated both the strength and completeness of evidence. LIHC is highlighted in red, whereas the other cancer types are shown in gray. Numerical labels above the bars indicate the integrated evidence scores, and fractions at the top indicate the number of available evidence domains.

For each cancer type, increased ATPSCKMT expression, association with adverse prognosis, and strong RNAi dependency were independently converted into directional percentile scores ranging from 0 to 100. Evidence in the opposite direction was assigned a score of zero, whereas unavailable evidence domains were left blank in Figure 5A. Labels ranging from 0/3 to 3/3 above each cancer type indicate the number of evaluable evidence domains, rather than the number of positive evidence domains. The composition of evidence varied among cancer types: some cancers had a high score in only one domain, whereas others had evidence from two or more domains.

In LIHC, all three domains—expression, prognosis, and RNAi dependency—were evaluable and showed relatively high directional scores (Figure 5A). The three domains were then integrated with equal weighting, with missing domains assigned a score of zero. LIHC had the highest integrated evidence score among all analyzed cancer types (81.5; Figure 5B), substantially exceeding the score for the second-ranked cancer type, UCEC (56.1). LUSC and SKCM followed with scores of 52.7 and 51.7, respectively. Thus, under the predefined scoring framework, LIHC had the highest integrated evidence score and was prioritized for subsequent analyses and experimental investigation of the cancer-related function of ATPSCKMT.

To further assess the robustness of LIHC prioritization and address the potential influence of missing evidence, we performed an additional complete-case analysis restricted to cancer types with evaluable expression, prognosis, and RNAi dependency data. Of the 51 cancer types, 13 had complete evidence, including both OS and PFI estimates for the prognosis domain. Directional percentile scores were recalculated exclusively within these 13 cancer types rather than retaining the scores derived from the original pan-cancer analysis. No missing evidence was imputed in this analysis (Supplementary Figure S3A). Under equal weighting of expression, prognosis, and RNAi dependency, LIHC retained the highest integrated evidence score (91.7), followed by UCEC (62.5) and LUSC (61.1; Supplementary Figure S3B).

Separately, we evaluated 13 alternative scoring scenarios across all 51 cancer types using the original pan-cancer scoring framework (Supplementary Figure S3C). LIHC consistently ranked first under the original equal-weighting model, all three alternative weighting schemes retaining expression, prognosis, and RNAi dependency, and the model assigning equal weights to expression, OS, PFI, and RNAi dependency. In contrast, single-indicator analyses yielded variable rankings, ranging from second for expression to tenth for RNAi dependency. Applying a nominal significance threshold of *p* < 0.05 to expression, OS, and PFI evidence preserved the first-place ranking of LIHC across the three-domain weighting scenarios (Supplementary Figure S3D). Together, these analyses support the robustness of LIHC prioritization under both the complete-case restriction and the evaluated pan-cancer weighting and significance-filtering conditions, while indicating that rankings may vary when individual evidence domains are considered separately.

### 2.6. ATPSCKMT Is Recurrently Upregulated in HCC and Supports HCC Cell Proliferation

**Figure 6:**
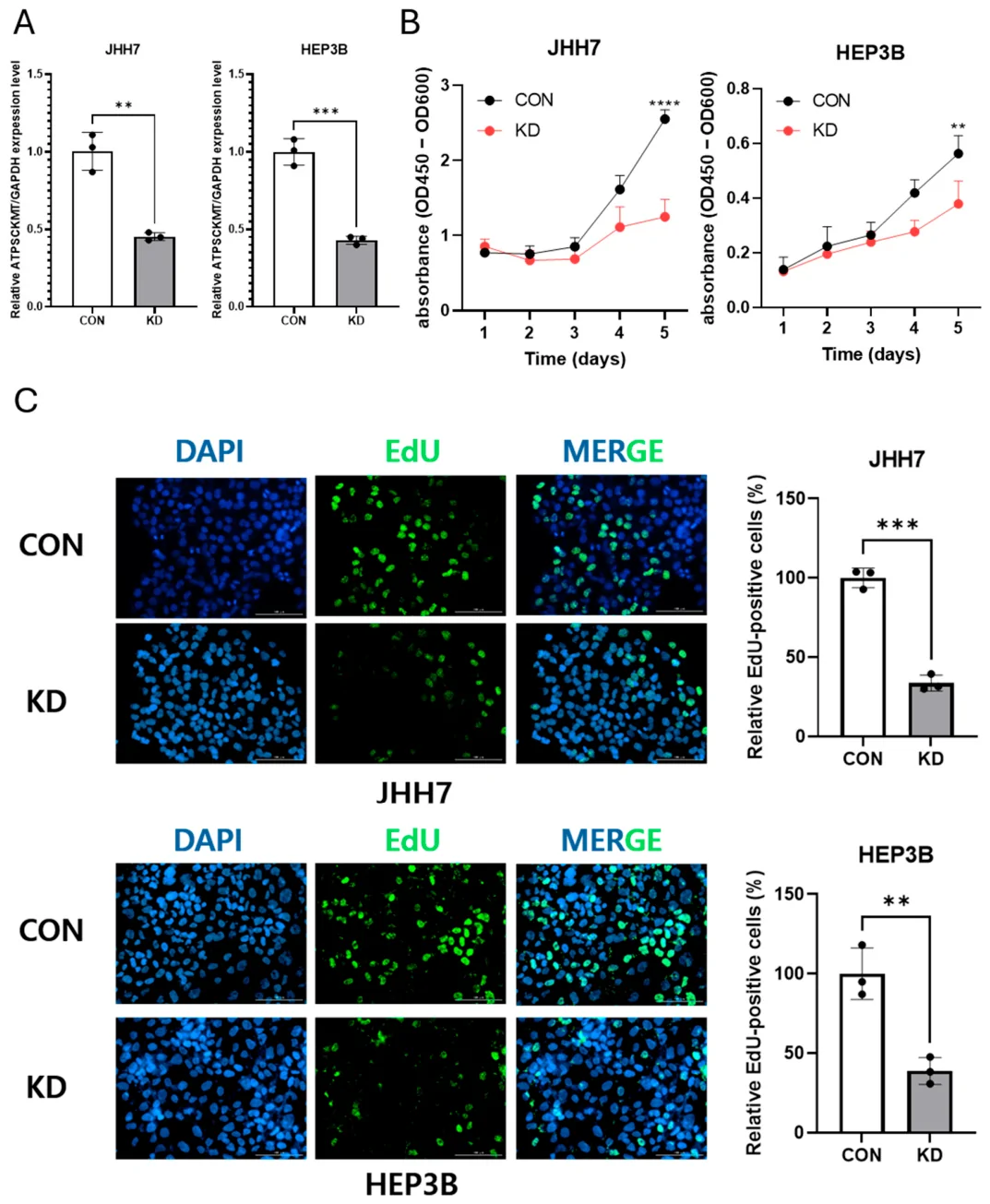
ATPSCKMT knockdown suppresses the proliferative capacity of hepatocellular carcinoma cells. (**A**) Relative ATPSCKMT mRNA expression in JHH7 and HEP3B cells following ATPSCKMT knockdown (KD), as determined by quantitative real-time PCR. ATPSCKMT expression was normalized to GAPDH and expressed relative to the control group (CON). (**B**) Cell viability of control and ATPSCKMT-KD JHH7 and HEP3B cells measured over five days using the CCK-8 assay. Corrected absorbance was calculated by subtracting the absorbance at 600 nm from that at 450 nm. (**C**) Representative fluorescence images and quantification of EdU incorporation in control and ATPSCKMT-KD JHH7 and HEP3B cells. EdU-positive cells are shown in green, and nuclei counterstained with DAPI are shown in blue. The proportion of EdU-positive cells was normalized to the corresponding control group. Scale bar = 100 μm. For qPCR and EdU assays, data are presented as the mean ± SD from three independent experiments (n = 3). For the CCK-8 assay, data are presented as the mean ± SD of six replicate wells per condition (n = 6) from a representative experiment, and the assay was independently repeated at least three times with comparable results. Statistical significance for qPCR and EdU assays was assessed using an unpaired two-tailed Student’s *t*-test. For the CCK-8 assay, control and ATPSCKMT-knockdown groups were compared at each time point using multiple unpaired *t*-tests, with multiple comparisons corrected using the two-stage linear step-up procedure of Benjamini, Krieger, and Yekutieli. ** *p* < 0.01, *** *p* < 0.001, and **** *p* < 0.0001. Scale bar: 100 µm.

Because the integrated pan-cancer analysis prioritized LIHC for further investigation of ATPSCKMT, we next evaluated the expression patterns of ATPSCKMT and the expanded METTL/7BS KMT gene set across multiple HCC cohorts [30]. In the integrated sample-level heatmap, METTL/7BS KMT genes displayed diverse expression patterns according to cohort and tissue type (Supplementary Figure S4A). Cohort-specific comparisons between tumor and adjacent non-tumor tissues showed recurrent upregulation of multiple METTL/7BS KMT genes in HCC. In particular, ATPSCKMT was significantly upregulated in multiple HCC cohorts, with no recurrent pattern of downregulation (Supplementary Figure S4B). These results indicate that the association between ATPSCKMT and LIHC identified in the pan-cancer analysis was reproducible across multiple HCC datasets and was not restricted to a single TCGA cohort. To assess the functional relevance of this recurrent upregulation, ATPSCKMT was knocked down in JHH7 and HEP3B cells. Quantitative real-time PCR confirmed that ATPSCKMT mRNA expression was reduced to less than half of the control level in both cell lines (Figure 6A). In the CCK-8 assay, the difference between the control and knockdown groups became more pronounced over time, and the absorbance of ATPSCKMT-knockdown cells was significantly reduced in both JHH7 and HEP3B cells on day 5 (Figure 6B). EdU-incorporation analysis similarly showed a marked reduction in the proportion of EdU-positive cells following ATPSCKMT knockdown in both cell lines (Figure 6C). Collectively, ATPSCKMT was recurrently upregulated across HCC cohorts, and its knockdown reduced both the CCK-8 signal and EdU incorporation in JHH7 and HEP3B cells. These findings support the possibility that ATPSCKMT contributes to maintenance of the proliferative phenotype of HCC cells and provide functional evidence for the ATPSCKMT-LIHC association identified through the integrated pan-cancer analysis.

### 2.7. ATPSCKMT Expression Is Recurrently Associated with Proliferation-Related Hallmark Programs

**Figure 7:**
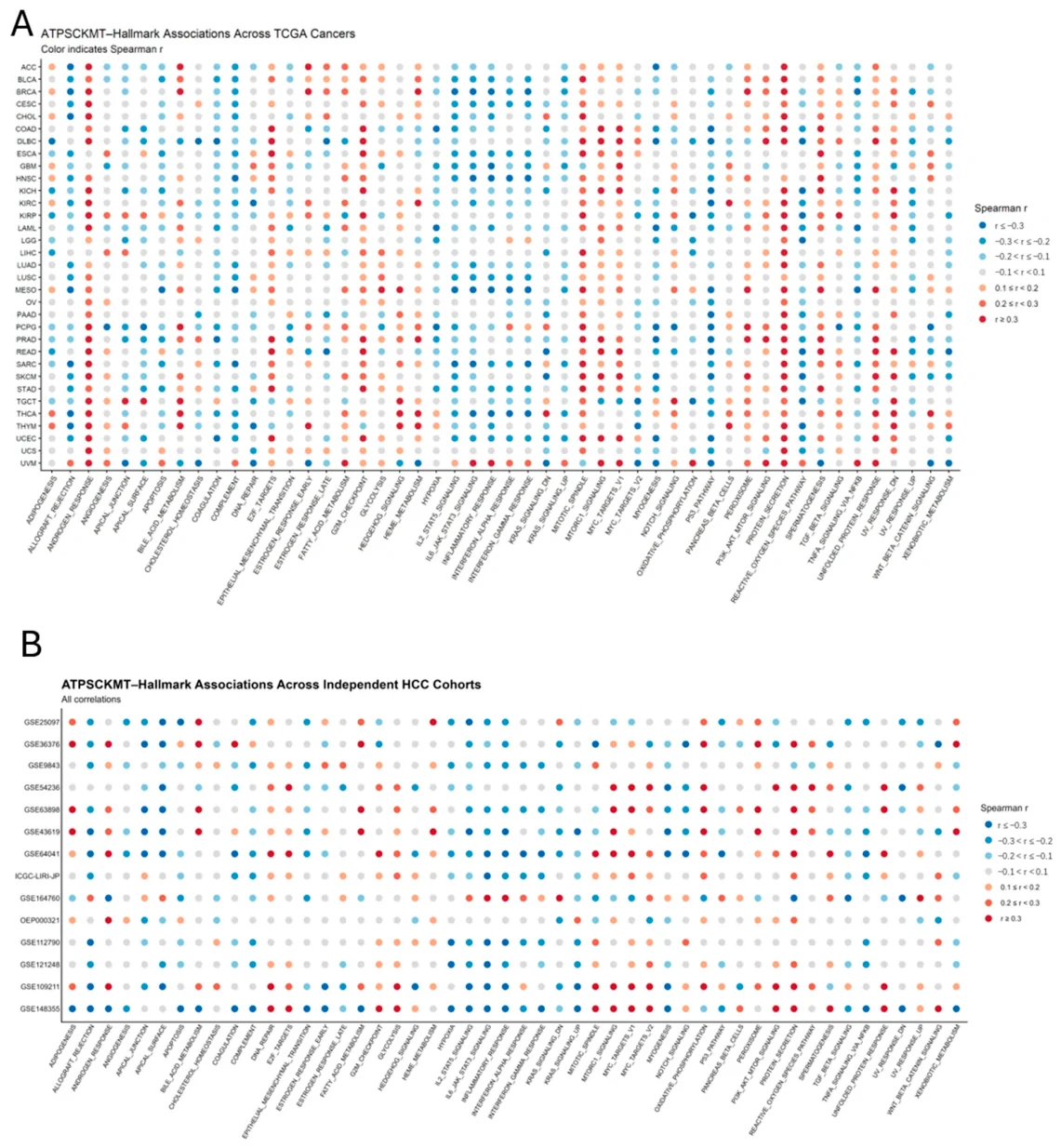
Pan-cancer and HCC cohort analyses of associations between ATPSCKMT expression and Hallmark gene-set activity. (**A**) Spearman correlations between ATPSCKMT expression and Hallmark gene-set scores across TCGA cancer types. (**B**) Spearman correlations between ATPSCKMT expression and Hallmark gene-set scores across 14 independent HCC cohorts, labeled by their original study accession. HCCDB15 (TCGA-LIHC) was excluded from the analysis. Colors indicate the direction and magnitude of the Spearman correlation coefficient, with red and blue representing positive and negative correlations, respectively, and gray representing weak correlations. All correlation coefficients are displayed irrespective of statistical significance. The proliferation-related signatures E2F TARGETS, G2M CHECKPOINT, MITOTIC SPINDLE, MYC TARGETS V1, and MYC TARGETS V2 are highlighted.

Given the reduction in proliferation-related readouts following ATPSCKMT knockdown in HCC cells, we next investigated molecular programs associated with ATPSCKMT expression. Spearman correlations between ATPSCKMT expression and Hallmark gene-set scores were analyzed across TCGA cancer types [31]. The correlation patterns varied among cancer types, indicating differences in ATPSCKMT-associated transcriptional features (Figure 7A). In TCGA-LIHC, ATPSCKMT expression was positively correlated with several proliferation-related signatures, including E2F TARGETS, G2M CHECKPOINT, MITOTIC SPINDLE, MYC TARGETS V1, and MYC TARGETS V2. These findings indicate that HCC tumors with higher ATPSCKMT expression tended to exhibit increased activity scores for transcriptional programs associated with cell-cycle progression and proliferation.

To evaluate the reproducibility of these associations, we performed the same analysis across 14 independent HCCDB cohorts, excluding HCCDB15 to avoid overlap with TCGA-LIHC [30]. Although the direction and magnitude of the correlations varied among cohorts, positive associations with E2F TARGETS, G2M CHECKPOINT, MITOTIC SPINDLE, and MYC TARGETS signatures were recurrently observed across multiple HCC datasets (Figure 7B). Thus, the association between ATPSCKMT expression and proliferation-related transcriptional programs was observed in both TCGA-LIHC and independent HCC cohorts. These results provide transcriptome-level evidence linking ATPSCKMT expression to proliferative molecular features in HCC.

### 2.8. Network and Multi-Cohort Co-Expression Analyses Identify Reproducible ATPSCKMT-Associated Genes in HCC

**Figure 8:**
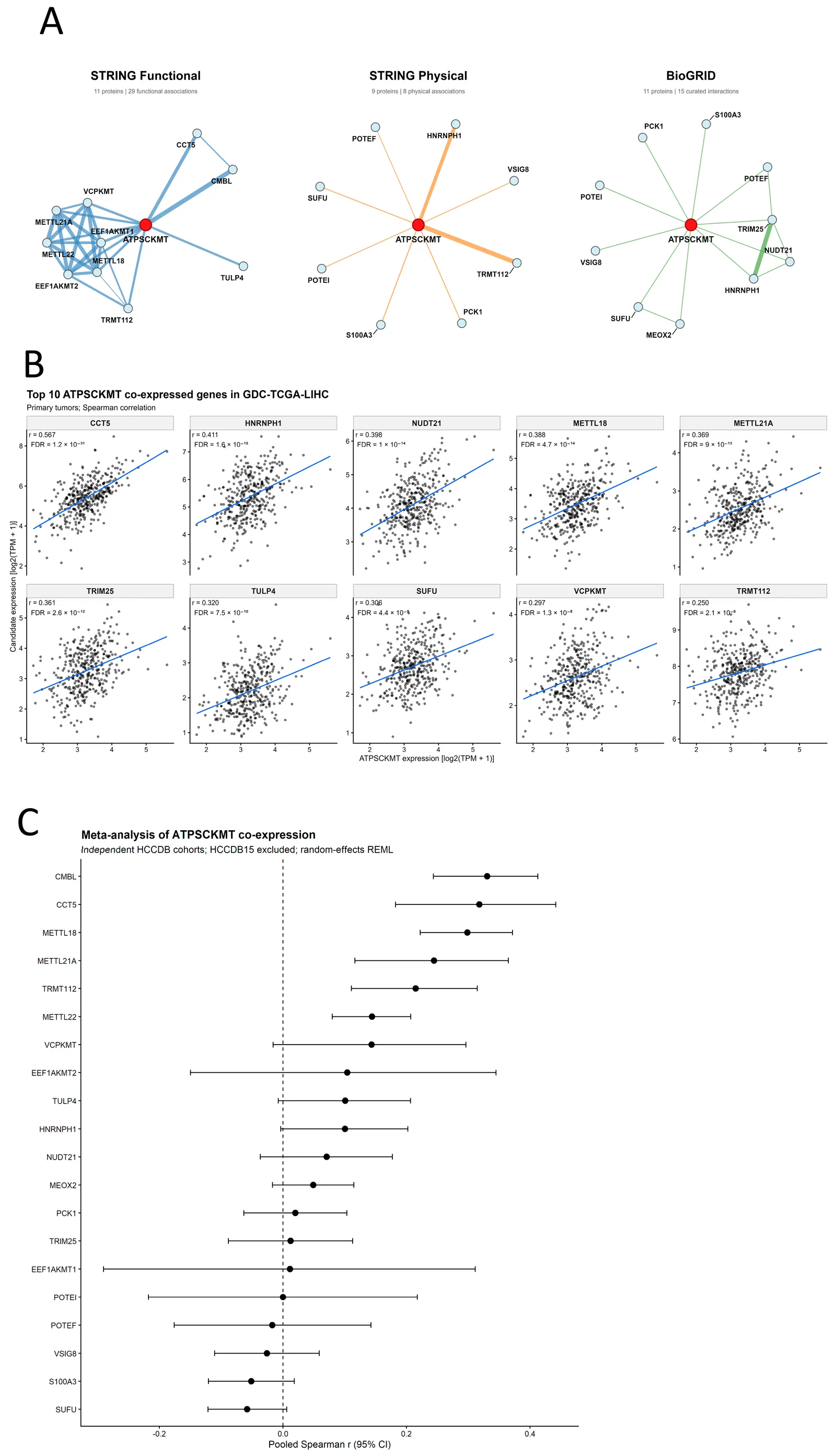
Integrated network and multi-cohort co-expression analyses of ATPSCKMT-associated genes in hepatocellular carcinoma. (**A**) ATPSCKMT-centered networks derived from STRING functional associations, STRING physical interactions, and curated BioGRID interactions. ATPSCKMT is shown as the central red node. Edge width represents the source-specific association score or number of supporting interaction records. Evidence metrics and edge widths are not directly comparable across the three databases. STRING functional associations do not necessarily represent direct physical interactions. (**B**) Scatter plots showing the ten candidate genes with the strongest positive Spearman correlations with ATPSCKMT expression in primary TCGA-LIHC tumors. Gene-expression values are presented as log2(TPM + 1). Spearman correlation coefficients (ρ) and FDR-adjusted *p* values are indicated in each panel. (**C**) Random-effects meta-analysis of correlations between ATPSCKMT and candidate-gene expression across independent HCCDB cohorts, excluding HCCDB15. Cohort-specific Spearman correlation coefficients were transformed using Fisher’s z transformation and pooled using a random-effects model with restricted maximum likelihood estimation. Pooled estimates were back-transformed to Spearman correlation coefficients. Points and horizontal lines indicate pooled correlation estimates and their 95% confidence intervals, respectively. The vertical dashed line indicates no correlation.

To identify ATPSCKMT-associated candidate genes, we constructed networks using STRING functional associations, STRING physical interactions, and BioGRID data [32,33]. After removing overlapping candidates, 20 non-redundant genes were retained (Figure 8A). In TCGA-LIHC primary tumors, CCT5 showed the strongest positive correlation with ATPSCKMT among the evaluated candidates (Spearman ρ = 0.567, FDR-adjusted *p* = 1.2 × 10^−31^; Figure 8B). Because ATPSCKMT and CCT5 are located in close genomic proximity at 5p15.2, we further examined their co-expression after adjustment for shared local copy-number variation. Among 362 TCGA-LIHC tumors with matched expression and ASCAT3 copy-number data, the correlation remained positive before (Spearman ρ = 0.563, *p* < 0.001) and after adjustment (partial Spearman ρ = 0.522, *p* < 0.001; Supplementary Figure S5).

To assess reproducibility, we performed a random-effects meta-analysis across 14 independent HCCDB cohorts, excluding HCCDB15. CMBL showed the highest pooled correlation (ρ = 0.331, 95% CI = 0.244–0.413), followed by CCT5 (ρ = 0.318, 95% CI = 0.182–0.442). METTL18, METTL21A, TRMT112, and METTL22 also showed significant positive pooled correlations after FDR correction (Figure 8C). These findings identified CCT5 as a reproducible ATPSCKMT co-expression candidate across independent HCC datasets.

### 2.9. ATPSCKMT-Positive Malignant Hepatocytes Exhibit Increased Detection of Multiple Candidate Genes

**Figure 9:**
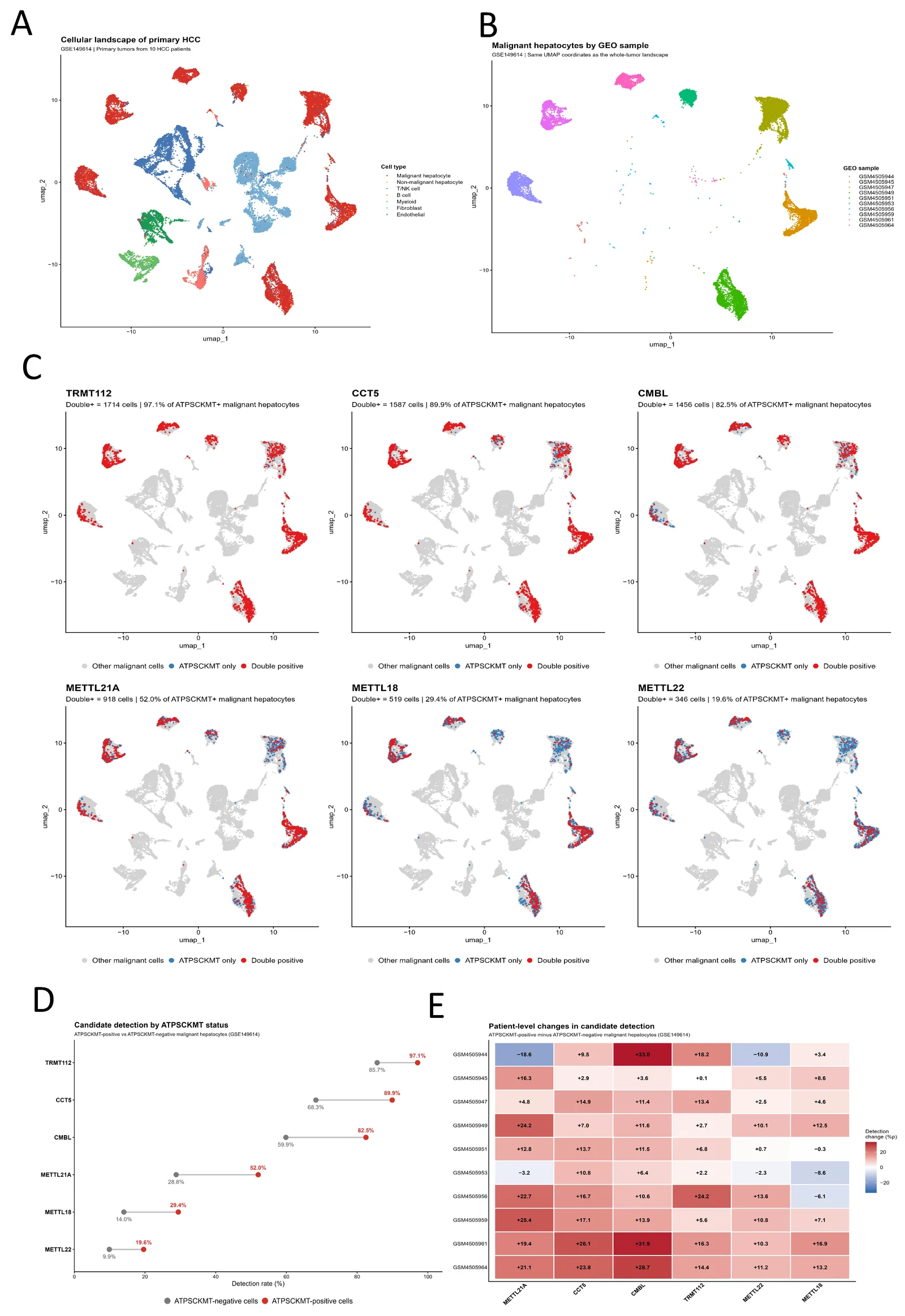
Single-cell characterization of ATPSCKMT and candidate gene detection in malignant hepatocytes from primary hepatocellular carcinoma. (**A**) UMAP representation of cells from primary HCC samples in GSE149614 from 10 patients, colored according to the indicated cell-type annotations. (**B**) UMAP representation of malignant hepatocytes colored according to the corresponding GEO sample accession. (**C**) UMAP representations of ATPSCKMT and candidate-gene detection in malignant hepatocytes for TRMT112, CCT5, CMBL, METTL21A, METTL18, and METTL22. Gray indicates ATPSCKMT-negative malignant cells, blue indicates cells with detectable ATPSCKMT but not the indicated candidate gene, and red indicates cells with detectable expression of both ATPSCKMT and the indicated candidate gene. The number of double-positive cells and the corresponding percentage among ATPSCKMT-positive malignant hepatocytes are shown above each plot. (**D**) Detection frequencies of the indicated candidate genes in ATPSCKMT-negative and ATPSCKMT-positive malignant hepatocytes. Gray and red points represent ATPSCKMT-negative and ATPSCKMT-positive cells, respectively. (**E**) Patient-level differences in candidate-gene detection between ATPSCKMT-positive and ATPSCKMT-negative malignant hepatocytes. Values represent percentage-point differences calculated within each sample as ATPSCKMT-positive minus ATPSCKMT-negative detection frequency.

To assess ATPSCKMT-associated candidate genes at the single-cell level, we analyzed the GSE149614 dataset comprising primary HCC samples from 10 patients [34]. A total of 13,691 malignant hepatocytes were identified (Figure 9A,B), of which 1765 cells (12.9%) showed detectable ATPSCKMT expression. The proportion of ATPSCKMT-positive malignant hepatocytes varied among patients, ranging from 5.8% to 24.6% (Supplementary Figure S6A).

Among ATPSCKMT-positive malignant hepatocytes, TRMT112 (97.1%), CCT5 (89.9%), and CMBL (82.5%) showed high co-detection frequencies, while METTL21A, METTL18, and METTL22 were detected at lower frequencies (Figure 9C). All six candidates were detected more frequently in ATPSCKMT-positive than in ATPSCKMT-negative cells (Figure 9D). For example, CCT5 was detected in 89.9% of ATPSCKMT-positive cells compared with 68.3% of ATPSCKMT-negative cells.

At the patient level, CCT5, CMBL, and TRMT112 showed higher detection frequencies in ATPSCKMT-positive cells in all 10 patients (Wilcoxon FDR = 0.0118 for each), whereas METTL21A showed the same direction in 8 of 10 patients (FDR = 0.0485). METTL18 and METTL22 did not reach statistical significance after FDR correction (Figure 9E).

However, ATPSCKMT-positive malignant hepatocytes showed higher median nCount_RNA (29,033 versus 12,736) and nFeature_RNA (4614 versus 2858) than ATPSCKMT-negative cells (both Wilcoxon *p* < 0.001). Therefore, differences in sequencing depth and transcript detection may contribute to the observed co-detection patterns, which should be interpreted as descriptive associations rather than evidence of direct regulation.

## 3. Discussion

In this study, we systematically investigated an expanded set of genes classified as members of the METTL family and/or seven-β-strand (7BS) lysine methyltransferases using complementary pan-cancer datasets. By integrating genomic alterations, differential expression, patient prognosis, and RNAi dependency, we prioritized ATPSCKMT for further investigation in hepatocellular carcinoma. Its association with HCC was subsequently examined using independent transcriptomic cohorts, cell-based functional assays, and single-cell analyses. Together, these complementary approaches provide evidence linking ATPSCKMT to proliferation-associated phenotypes and molecular features in HCC.

Although previous studies have investigated individual METTL enzymes and their cancer-associated functions, systematic comparisons incorporating structurally related non-METTL 7BS lysine methyltransferases remain limited [21]. Our findings revealed heterogeneous genomic alteration patterns, expression changes, and prognostic associations across the expanded gene set. These differences are consistent with the diverse substrate specificities and biological functions of the included methyltransferases, which should not be assumed to act through a shared molecular mechanism. Moreover, prognostic associations derived from pooled pan-cancer analyses require cautious interpretation because cancer-type heterogeneity and relatively small mutation-defined subgroups may influence survival estimates.

The integrated prioritization framework was designed to identify cancer types supported by convergent evidence rather than to generate a clinical prediction score. In the original pan-cancer analysis, LIHC achieved the highest integrated evidence score among 51 cancer types, and its leading position was preserved across alternative weighting schemes that jointly incorporated expression, prognosis, and RNAi dependency, including after significance filtering (Supplementary Figure S3C,D). To address the potential influence of missing evidence, we additionally performed a complete-case analysis restricted to 13 cancer types with evaluable data in all three domains. Domain-specific percentile scores and integrated rankings were recalculated within this subset without imputing missing evidence. LIHC retained the highest integrated score among these 13 cancer types (Supplementary Figure S3A,B). These complementary analyses strengthen the robustness of LIHC prioritization under the evaluated scoring conditions. Nevertheless, the integrated prioritization framework remains exploratory and has not been independently validated as a quantitative measure of biological importance. The observed robustness does not establish the biological validity of the selected indicators, their relative weights, or the resulting composite score. Furthermore, the original assignment of zero to unavailable evidence should be distinguished from a zero score resulting from an observed effect in the opposite direction. Missing evidence does not imply the biological absence of an association, and the resulting cancer-type rankings remain dependent on evidence availability, scoring procedures, and the composition of the comparison set. Accordingly, the integrated framework should be regarded as a hypothesis-generating approach for prioritizing cancer types for further investigation of ATPSCKMT rather than as a validated measure of cancer-type-specific biological dependency or therapeutic relevance.

An important finding was the discrepancy between the RNAi- and CRISPR-based dependency profiles of ATPSCKMT. DEMETER2 identified widespread sensitivity to ATPSCKMT knockdown, whereas Chronos did not indicate comparable dependency under the applied descriptive threshold. These platforms differ in perturbation modality, cell-line coverage, screening conditions, and dependency models, which may contribute to the divergent results. However, the underlying reasons for this discrepancy remain unresolved. Importantly, the integrated prioritization framework relied on RNAi-derived dependency scores, and the discrepancy with CRISPR-based results warrants caution when interpreting the broader applicability of the observed RNAi dependency. Therefore, RNAi- and CRISPR-based dependency results should be interpreted as distinct, non-interchangeable lines of evidence regarding ATPSCKMT dependency.

The cellular experiments provided additional support for the association between ATPSCKMT and proliferation-related phenotypes in HCC. ATPSCKMT knockdown reduced CCK-8 signals and EdU incorporation in both JHH7 and HEP3B cells. Because CCK-8 reflects cellular metabolic activity as well as viable cell number, whereas EdU incorporation measures DNA synthesis, these results are consistent with reduced proliferation-associated activity following ATPSCKMT depletion. However, EdU incorporation does not directly measure mitotic activity, and the mitotic index was not determined in this study. Therefore, these findings do not establish whether ATPSCKMT depletion specifically affects mitotic progression, cell survival, or mitochondrial metabolism. ATPSCKMT is known to methylate the mitochondrial ATP synthase c-subunit and influence ATP synthase function [22,23]. Given the importance of metabolic regulation in cancer-cell proliferation and survival [35], this established biochemical function provides a rationale for investigating the potential involvement of ATPSCKMT in cancer-cell metabolism. Nevertheless, mitochondrial respiration, ATP production, and substrate methylation were not directly evaluated in the present HCC models, and the observed phenotype cannot be attributed specifically to mitochondrial ATP synthase dysfunction.

Consistent with these cellular findings, ATPSCKMT expression was positively associated with proliferation-related Hallmark gene-set scores in TCGA-LIHC and multiple independent HCC cohorts. E2F and MYC are important regulators of transcriptional programs involved in cell-cycle entry, DNA replication, and cellular proliferation [36,37]. Associations with G2M CHECKPOINT and MITOTIC SPINDLE signatures further suggest that high ATPSCKMT expression occurs in tumors exhibiting transcriptional features related to cell-cycle progression. However, ssGSEA-based correlations describe molecular states associated with ATPSCKMT expression and do not demonstrate direct activation or regulation of these pathways by ATPSCKMT.

Among the network-derived candidates, CCT5 emerged as a representative gene associated with ATPSCKMT expression across multiple HCC datasets. Importantly, ATPSCKMT and CCT5 are located in close genomic proximity at chromosome 5p15.2, raising the possibility that shared regional copy-number alterations contribute to their observed co-expression. Although the association remained positive after adjustment for shared local copy-number variation, this finding does not exclude other common transcriptional or cellular influences. Similarly, the reproducibility of the association across independent HCC cohorts supports its consistency but does not establish a direct regulatory or functional relationship between the two genes.

CCT5 encodes one of the eight subunits of the ATP-dependent chaperonin-containing TCP-1 (CCT/TRiC) complex, which facilitates the folding of proteins including actin and tubulin [38,39]. Through these functions, the CCT/TRiC complex contributes to cytoskeletal organization and processes required for mitotic progression. CCT5 is also included in the HALLMARK_MYC_TARGETS_V1 gene set [31], providing a possible transcriptional context for its association with proliferation-related programs. Previous studies in HCC have reported that CCT5 expression influences cell proliferation, colony formation, and cell-cycle progression [40]. These established functions suggest that CCT5 is biologically relevant to proliferative processes in HCC. Nevertheless, the present findings do not demonstrate that CCT5 mediates the proliferation-associated phenotype observed following ATPSCKMT knockdown, nor do they establish an ATPSCKMT–CCT5 regulatory axis.

Single-cell analysis provided additional descriptive evidence that ATPSCKMT and several candidate genes can be detected within the same malignant hepatocytes. The patient-level consistency observed for CCT5, CMBL, and TRMT112 suggests that these associations are not restricted to a pooled cell population. However, ATPSCKMT-positive malignant hepatocytes also showed substantially higher transcript-detection depth, and single-cell gene detection is influenced by library complexity and dropout [41]. Although restricting the analysis to malignant hepatocytes reduces the influence of non-malignant cell composition, the observed co-detection patterns cannot establish specific co-regulation or direct functional relationships. Therefore, the single-cell results should be viewed as supporting the prioritization of candidate genes for subsequent investigation rather than providing mechanistic validation.

Several limitations should be acknowledged. First, the genomic, transcriptomic, and survival analyses were largely based on retrospective public datasets and cannot establish causality. Clinical heterogeneity and unmeasured confounders may influence the observed prognostic associations. Second, although the complete-case analysis avoided imputing unavailable evidence and LIHC remained the top-ranked cancer type within the eligible subset, this analysis was restricted to 13 cancer types and may be subject to selection effects related to data availability. Other missing-data approaches, such as available-case weighting or statistical imputation, were not systematically evaluated. Third, functional validation was limited to siRNA-mediated knockdown in two HCC cell lines, without direct assessment of mitochondrial enzyme activity, ATP production, substrate methylation, hypoxia-related signaling such as HIF-1α, or functional rescue. Thus, the molecular mechanism underlying the observed proliferation-associated phenotype and its potential relationship with mitochondrial metabolism and hypoxia-related pathways remain unresolved. Finally, the single-cell findings were derived from one patient cohort and may be affected by differences in transcript detection. Future studies incorporating independent perturbation approaches, biochemical measurements, and functional rescue experiments will be necessary to clarify the contribution of ATPSCKMT to HCC proliferation and its potential functional relationships with candidates such as CCT5.

## 4. Materials and Methods

### 4.1. Definition and Classification of the METTL/7BS KMT Analysis Set

An expanded methyltransferase analysis set was constructed to include genes classified as members of the methyltransferase-like (METTL) family and/or as seven-β-strand lysine methyltransferases (7BS KMTs). The final analysis set comprised 38 genes, which were classified into three groups: METTL family only (n = 24), METTL family + 7BS KMT (n = 9), and 7BS KMT only (n = 5). Thus, the analysis set contained 33 METTL family genes and 14 7BS KMT genes, with nine genes shared between the two classifications [9,12]. Current gene symbols and representative former or alternative symbols were verified using the HUGO Gene Nomenclature Committee (HGNC) database [42]. Classification as a 7BS lysine methyltransferase was based on available structural and functional annotations. Chromosomal locations were reported as cytogenetic bands based on the human GRCh38 assembly. UniProtKB canonical human protein entries were used to obtain accession numbers, amino-acid sequence lengths, and molecular weights [43]. The complete classification and molecular characteristics of the 38 genes are summarized in Table 1.

### 4.2. Pan-Cancer Genomic Alteration Analysis

Genomic alteration data for the METTL/7BS KMT gene set were obtained from the TCGA PanCancer Atlas datasets available through cBioPortal (https://www.cbioportal.org/; accessed on 26 February 2026) [25,26]. Somatic mutations, structural variants, copy-number amplifications, deep deletions, and multiple alterations were evaluated across TCGA cancer types. Alteration frequencies were summarized according to cancer type and gene. For genes showing relatively high amplification frequencies, discrete copy-number alteration data and corresponding mRNA expression data were used to examine the relationship between copy-number status and gene expression. In the survival analyses, samples with a discrete copy-number value of +2 were classified as amplified, whereas profiled samples without amplification were classified as non-amplified. Samples not profiled for copy-number alterations were excluded. For mutation-associated analyses, patients were classified according to the presence or absence of non-silent somatic mutations in the corresponding gene. Mutation locations across protein sequences were additionally examined for selected genes. Overall survival (OS) was compared between mutated and non-mutated patient groups using Kaplan–Meier analysis, and statistical differences were assessed using the log-rank test. Patient-level clinical information provided with the TCGA PanCancer Atlas datasets was used for survival analyses.

### 4.3. Pan-Cancer Differential Expression Analysis

Gene-level RNA-sequencing data generated using the STAR-Counts workflow were obtained from the National Cancer Institute Genomic Data Commons (GDC; https://portal.gdc.cancer.gov/; accessed on 10 August 2026) [27]. TPM values provided in the GDC gene-expression quantification files (tpm_unstranded) were extracted, and primary tumor and solid tissue normal samples were identified according to TCGA sample-type annotations. Only cancer types containing at least three primary tumor samples and three solid tissue normal samples were included in tumor-versus-normal comparisons. For each METTL/7BS KMT gene and cancer type, differences in TPM values between tumor and normal tissues were evaluated using the Wilcoxon rank-sum test. Median expression was calculated separately for tumor and normal samples, and log2 fold change was calculated as log2FC = log2[(median TPM_tumor + 0.01)/(median TPM_normal + 0.01)]. *p*-values were adjusted using the Benjamini–Hochberg method within each cancer type. Differential expression was considered significant when the false discovery rate (FDR) was <0.05 and the absolute log2 fold change was ≥1. Cancer types for which either tumor or normal samples did not meet the minimum sample requirement were excluded from the corresponding comparison.

### 4.4. Pan-Cancer Survival Analysis

Pan-cancer survival analyses were performed by integrating GDC-derived gene-expression data with clinical outcome information from the TCGA Pan-Cancer Clinical Data Resource (TCGA-CDR) [28]. Gene expression was analyzed as a continuous variable after log2(TPM + 1) transformation. Cox proportional hazards regression was performed separately for each METTL/7BS KMT gene and cancer type. Overall survival (OS) and progression-free interval (PFI) were used as clinical endpoints. Univariate Cox regression models were first fitted using gene expression as the explanatory variable. Multivariable Cox regression was subsequently performed using gene expression, age, and sex as covariates. Hazard ratios (HRs), 95% confidence intervals (CIs), and *p*-values were calculated for each gene–cancer combination. *p*-values were adjusted using the Benjamini–Hochberg method separately for each survival endpoint in the univariate analyses and for each model–endpoint combination in the multivariable analyses. HR > 1 indicated an association between higher expression and increased risk, whereas HR < 1 indicated an association with reduced risk. Samples lacking the required expression, survival, age, or sex information were excluded from the corresponding models. FDR < 0.05 was considered statistically significant.

### 4.5. Cancer Dependency Analysis

RNA interference (RNAi) gene dependency data were obtained from the DepMap portal (https://depmap.org/portal/; accessed on 10 August 2025). The primary dependency analysis used the DEMETER2-integrated RNAi dataset [29], which combines RNAi screening data from the Achilles, DRIVE, and Marcotte projects. In addition, CRISPR–Cas9 gene-effect scores from DepMap Public 25Q2+Score, processed using the Chronos model [44], were analyzed in parallel as an independent perturbation modality. Cell-line cancer-type annotations were assigned using the corresponding DepMap model classifications. For each platform separately, a gene–cancer type combination was considered evaluable only when at least five cell lines had non-missing dependency scores. Mean dependency scores were then calculated for each evaluable gene–cancer type combination. A mean DEMETER2 score < −0.3 was used as the predefined criterion for RNAi dependency. For descriptive consistency across the two platforms, a mean Chronos score < −0.3 was used to summarize cancer-type dependency patterns. Because DEMETER2 and Chronos are derived from distinct perturbation platforms and dependency models, scores from the two datasets were interpreted within each platform rather than as directly interchangeable measurements. Combinations with fewer than five non-missing cell lines were treated as unavailable rather than as non-dependent observations. Of the 38 genes in the expanded analysis set, 20 were represented in the DEMETER2 dataset and all 38 were represented in the Chronos dataset. Because DEMETER2 provided discriminating variation across the expanded gene set, it was used for the integrated cancer-type prioritization; the CRISPR/Chronos results are reported in parallel and were not incorporated into the prioritization score.

### 4.6. Integrated Pan-Cancer Prioritization of ATPSCKMT

To prioritize cancer types for subsequent investigation of ATPSCKMT, three evidence domains were integrated: tumor-versus-normal expression, patient prognosis, and RNAi dependency. In the original pan-cancer analysis, tumor-versus-normal log2 fold-change values were converted to percentile scores among cancer types with evaluable expression data. For the prognosis domain, log-transformed hazard ratios from univariate overall survival (OS) and progression-free interval (PFI) Cox regression analyses were separately converted to percentile scores, and their arithmetic mean was used as the prognosis score. In the original scoring framework, the prognosis domain was considered evaluable when at least one of the two survival endpoints was available; an unavailable endpoint contributed zero to the combined prognosis score. Age- and sex-adjusted multivariable Cox regression results were used as secondary robustness evidence and were not incorporated into the integrated score.

For the RNAi dependency domain, negative mean DEMETER2 scores were converted to percentile scores, with greater dependency corresponding to higher scores. At least five cell lines with non-missing DEMETER2 values were required for RNAi evaluation. Evidence in the opposite direction was assigned a directional score of zero. In Figure 5A, unavailable evidence domains were left blank to distinguish missing data from evaluable evidence. For the original integrated pan-cancer score, unavailable domains contributed zero. Expression, prognosis, and RNAi dependency scores were equally weighted, and their arithmetic mean was used to calculate the integrated evidence score. The number of evaluable domains was also indicated to represent evidence completeness. The parallel CRISPR/Chronos analysis was reported separately and was not incorporated into the prioritization score.

To examine the influence of missing evidence, an additional complete-case analysis was performed. Only cancer types with evaluable tumor-versus-normal expression, both OS and PFI hazard-ratio estimates, and RNAi dependency data from at least five cell lines were retained. Thirteen of the original 51 cancer types satisfied these criteria. The complete-case subset was defined before recalculating the evidence scores. Directional percentile scores for expression, OS, PFI, and RNAi dependency were independently recalculated using only these 13 cancer types and the percent-rank method. As in the original framework, tumor-associated upregulation, adverse prognostic associations, and stronger RNAi dependency contributed positive directional percentile scores, whereas observed effects in the opposite direction received zero scores. Missing values were neither imputed nor assigned zero in the complete-case analysis. The recalculated OS and PFI scores were averaged to obtain the prognosis score, and the expression, prognosis, and RNAi dependency scores were equally weighted to generate the integrated evidence score. Cancer-type rankings were then recalculated within the 13-cancer subset.

Separately, a post hoc sensitivity analysis was performed across the original 51 cancer types to examine the influence of alternative weighting schemes. Thirteen scoring scenarios were evaluated: the original three-domain equal-weighting model; three alternative three-domain models assigning 50% weight to one domain and 25% to each remaining domain; four single-indicator models (expression, OS, PFI, and RNAi dependency); four pairwise models (OS and PFI, expression and prognosis, prognosis and RNAi dependency, and expression and RNAi dependency), with equal weighting within each pair; and one model assigning equal weights to expression, OS, PFI, and RNAi dependency as four individual indicators (25% each). A parallel sensitivity analysis applied a nominal *p*-value threshold of 0.05 to expression, OS, and PFI evidence while retaining the original RNAi scores. In these full pan-cancer sensitivity analyses, unavailable evidence continued to contribute zero. LIHC scores and rankings were compared across the 13 scoring scenarios, with all rankings determined among the original 51 cancer types.

### 4.7. HCCDB Cohort Processing and Independent HCC Validation

Independent HCC transcriptomic datasets were obtained from the HCCDB database (http://lifeome.net/database/hccdb/; accessed on 7 April 2026) [30]. Processed gene-expression matrices and corresponding sample annotations provided by HCCDB were used.

HCC and non-tumor liver samples were identified according to the sample annotations supplied for each cohort. Cohorts or samples lacking expression information for the genes required for a specific analysis were excluded from that analysis. HCCDB15 was excluded from independent validation analyses because it contains TCGA-LIHC samples and therefore overlaps with the GDC-TCGA-LIHC cohort. For analyses of gene-expression relationships, only HCC samples were retained. Expression analyses were performed separately within each cohort to avoid direct comparison of expression values across platforms. The independent HCCDB cohorts were subsequently used for pathway-association analyses and validation of ATPSCKMT-associated gene co-expression relationships as described below. For cohort-specific differential-expression analyses, HCC samples were compared with adjacent non-tumor liver samples when both groups were available. Differential expression was evaluated separately within each cohort using linear models implemented in the limma package [45], followed by empirical Bayes moderation. Log2 fold changes and Benjamini–Hochberg-adjusted *p*-values were obtained for each gene. Expression values were not directly combined across cohorts because of differences in the original study platforms and preprocessing procedures. For the integrated sample-level heatmap, expression values were standardized within each gene before visualization.

### 4.8. Hallmark ssGSEA Analysis

Hallmark gene sets were obtained from the Molecular Signatures Database (MSigDB; accessed on 13 August 2026) using the msigdbr R package [31]. Single-sample gene set enrichment analysis (ssGSEA) scores were calculated using the GSVA package in R [46]. For GDC data, Ensembl gene identifiers were mapped to gene symbols, and expression values corresponding to duplicated gene symbols were averaged. TPM values were transformed as log2(TPM + 1) before ssGSEA. For HCCDB cohorts, gene symbols provided in the processed expression matrices were used for gene-set matching. Hallmark gene sets represented by fewer than five genes in a given dataset were excluded. ssGSEA scores were calculated independently for each sample. GDC-TCGA cancer types with fewer than 20 evaluable tumor samples and HCCDB cohorts with fewer than 20 evaluable HCC samples were excluded from the corresponding correlation analysis. Associations between continuous ATPSCKMT expression and individual Hallmark ssGSEA scores were assessed using Spearman rank correlation. The analysis was performed across GDC-TCGA cancer types and independently repeated across HCCDB cohorts, with HCCDB15 excluded. *p*-values were adjusted using the Benjamini–Hochberg method separately within each cancer type or cohort.

### 4.9. Protein Interaction, Multi-Cohort Co-Expression, and Copy-Number-Adjusted Analysis

ATPSCKMT-associated proteins were identified using the STRING database (https://string-db.org/; accessed on 13 August 2026) and the BioGRID database (https://thebiogrid.org/; accessed on 13 August 2026) [32,33]. STRING functional-association and physical-interaction networks were evaluated separately. Gene symbols were harmonized before integration, including conversion of the former ATPSCKMT symbol FAM173B to ATPSCKMT. The candidate set comprised all 10 BioGRID interaction partners, all 8 STRING physical-interaction partners, and the top 10 STRING functional-association partners ranked according to the STRING functional score. After harmonization of gene symbols and removal of duplicated genes across databases, 20 non-redundant candidate genes were retained for downstream co-expression analysis. Co-expression between ATPSCKMT and each network-derived candidate was first assessed in GDC-TCGA-LIHC primary tumors. Only primary tumor samples were included, and where multiple primary tumor aliquots were available for the same patient, a single sample was retained. Expression values were transformed as log2(TPM + 1), and Spearman rank correlation coefficients and corresponding *p*-values were calculated. *p*-values were adjusted using the Benjamini–Hochberg method, and candidates were ranked according to their Spearman correlation coefficients. Reproducibility of the ATPSCKMT–candidate co-expression relationships was subsequently evaluated across independent HCCDB cohorts. HCCDB15 was excluded to prevent overlap with TCGA-LIHC. Within each cohort, Spearman correlation coefficients were calculated using HCC samples. Candidate–cohort combinations with fewer than 20 complete observations were excluded. *p*-values were adjusted using the Benjamini–Hochberg method across evaluable candidates within each cohort. For each candidate, cohort-specific Spearman correlation coefficients were transformed using Fisher’s z transformation and combined using a random-effects meta-analysis with restricted maximum likelihood (REML) estimation. At least two eligible cohorts were required for meta-analysis. Pooled correlation estimates and 95% confidence intervals were calculated after back-transformation. Between-cohort heterogeneity was evaluated using Cochran’s Q statistic, I^2^, and τ^2^. Meta-analysis-derived *p*-values were corrected using the Benjamini–Hochberg method. Because ATPSCKMT and CCT5 are located in close genomic proximity on chromosome 5p15.2, an additional analysis was performed to assess whether their RNA-level co-expression could be explained by shared local copy-number variation. Gene-level copy-number estimates for TCGA-LIHC were obtained from the GDC ASCAT3 workflow and matched to the expression data from primary tumors by patient identifier [47]. cBioPortal-derived discrete GISTIC copy-number calls were used for the pan-cancer alteration and amplification analyses to provide harmonized copy-number classifications across TCGA cancer types, whereas GDC ASCAT3 gene-level copy-number estimates were used specifically for the TCGA-LIHC ATPSCKMT–CCT5 analysis to enable patient-level matching with the GDC RNA-sequencing cohort and adjustment for local copy-number variation. Only tumors with complete ATPSCKMT expression, CCT5 expression, and gene-level copy-number data were included in the analysis. Because the ATPSCKMT and CCT5 gene-level copy-number variables were perfectly collinear in the matched dataset, a single shared local copy-number variable was used to avoid multicollinearity. ATPSCKMT expression, CCT5 expression, and the shared copy-number variable were rank-transformed. Ranked ATPSCKMT and CCT5 expression values were separately regressed against the ranked shared copy-number variable, and Pearson correlation between the resulting residuals was used to estimate the partial Spearman correlation. The unadjusted Spearman correlation was calculated using the same matched tumors for direct comparison. Ninety-five percent confidence intervals were estimated using Fisher’s z transformation.

### 4.10. Single-Cell RNA-Sequencing Analysis

Single-cell RNA-sequencing data were obtained from the Gene Expression Omnibus (GEO) under accession number GSE149614 (accessed on 14 August 2026) [34]. The authors’ processed cell metadata (GSE149614_HCC.metadata.updated.txt.gz) and gene-by-cell count matrix (GSE149614_HCC.scRNAseq.S71915.count.txt.gz) were downloaded from the GEO repository. Primary HCC samples from 10 patients were included. Cell-type annotations supplied with the dataset were used to identify the major cellular populations, and malignant hepatocytes were subsequently extracted for candidate-gene analyses. A total of 13,691 malignant hepatocytes were retained. Six network-derived candidates (TRMT112, CCT5, CMBL, METTL18, METTL21A, and METTL22) were evaluated in the single-cell validation analysis. Single-cell data were processed using the Seurat package in R [48]. The same UMAP coordinates generated for the whole primary-HCC cellular landscape were retained when malignant hepatocytes were visualized separately; UMAP was not recalculated for candidate-gene co-detection analyses. Gene detection was defined using raw RNA counts, with a gene considered detected when its raw count was >0. Because ATPSCKMT was represented by its former symbol FAM173B in some expression matrices, both symbols were checked during gene matching. ATPSCKMT-positive malignant hepatocytes were defined as cells with detectable ATPSCKMT/FAM173B transcripts, whereas ATPSCKMT-negative malignant hepatocytes lacked detectable transcripts. Among 13,691 malignant hepatocytes, 1765 were classified as ATPSCKMT-positive and 11,926 as ATPSCKMT-negative. For each of the six candidate genes, detection frequencies were compared between ATPSCKMT-positive and ATPSCKMT-negative malignant hepatocytes without subsampling. Pooled cell-level comparisons were evaluated using Fisher’s exact test, followed by Benjamini–Hochberg correction across the six candidates. Candidate-gene detection frequencies were additionally calculated separately for each of the 10 patients, and paired differences between ATPSCKMT-positive and ATPSCKMT-negative malignant hepatocytes were evaluated using the Wilcoxon signed-rank test. To assess potential confounding by cell-specific sequencing depth, total RNA counts (nCount_RNA) and the number of detected genes (nFeature_RNA) were compared between ATPSCKMT-positive and ATPSCKMT-negative malignant hepatocytes using the Wilcoxon rank-sum test.

### 4.11. Cell Culture and siRNA Transfection

Human hepatocellular carcinoma cell lines JHH7 and HEP3B were kindly provided by Professor Sae-Ock Oh. JHH7 cells were cultured in Williams’ Medium E (WELGENE, Gyeongsan, Republic of Korea; Cat. No. LM017-02), whereas HEP3B cells were cultured in high-glucose Dulbecco’s modified Eagle’s medium (DMEM; WELGENE; Cat. No. LM001-05). Culture media were supplemented with 10% fetal bovine serum and 1% penicillin–streptomycin. Cells were maintained at 37 °C in a humidified atmosphere containing 5% CO_2_. For ATPSCKMT knockdown, cells were transfected with pooled siRNAs targeting ATPSCKMT or a non-targeting control siRNA using Lipofectamine RNAiMAX Transfection Reagent (Invitrogen, Thermo Fisher Scientific, Waltham, MA, USA) according to the manufacturer’s instructions. The final siRNA concentration was 100 nM. For gene-expression analysis, cells were harvested 48 h after siRNA transfection. Cell-based functional assays were performed at the indicated time points following transfection. The siRNA sequences used in this study are provided in Supplementary Table S1.

### 4.12. RNA Extraction and Real-Time PCR

Cells were harvested 48 h after siRNA transfection, and total RNA was extracted using TRIzol Reagent (Invitrogen, Thermo Fisher Scientific, Waltham, MA, USA) according to the manufacturer’s instructions. Complementary DNA was synthesized using the PrimeScript™ 1st strand cDNA Synthesis Kit (Takara Bio, Shiga, Japan; Cat. No. 6110A). Quantitative real-time PCR was performed using Power SYBR™ Green PCR Master Mix (Applied Biosystems, Thermo Fisher Scientific; Cat. No. 4367659). Relative ATPSCKMT expression was calculated using the 2^−ΔΔCt^ method, with GAPDH as the internal reference gene. Primer sequences are provided in Supplementary Table S2. Each biological sample was measured in technical triplicate, and three independent experiments were performed.

### 4.13. CCK-8 Cell Viability Assay

Cell viability was assessed using D-Plus™ CCK Cell Viability Assay Reagent (Dongin LS, Seoul, Republic of Korea; Cat. No. CCK-3000). JHH7 and HEP3B cells were seeded into 96-well plates at a density of 2 × 10^3^ cells per well. Cell viability measurements were initiated 24 h after siRNA transfection. At each indicated time point, CCK reagent was added according to the manufacturer’s instructions, and cells were incubated for 1 h at 37 °C. Absorbance was measured at 450 and 600 nm using a microplate reader, and corrected absorbance was calculated by subtracting the absorbance at 600 nm from that at 450 nm. Six replicate wells were analyzed for each condition in each experiment. The assay was independently repeated at least three times, with comparable results obtained across independent experiments. Data from a representative experiment are presented as the mean ± SD.

### 4.14. EdU Incorporation Assay

Cell proliferation was evaluated using the Click-iT™ Plus EdU Cell Proliferation Kit for Imaging with Alexa Fluor™ 488 dye (Invitrogen, Thermo Fisher Scientific; Cat. No. C10637). Following siRNA transfection, cells were incubated with EdU at a final concentration of 5 μM for 2 h at 37 °C. Cells were subsequently fixed, permeabilized, and subjected to click-chemistry-based fluorescent labeling according to the manufacturer’s instructions. Cell nuclei were counterstained with DAPI. Fluorescence images were acquired using a BioTek Lionheart™ FX Automated Microscope B (BioTek Instruments, Winooski, VT, USA; Agilent Technologies, Santa Clara, CA, USA). EdU-positive cells and total DAPI-positive nuclei were quantified, and the percentage of EdU-positive cells was calculated for each condition. Three replicate wells were analyzed per condition in each independent experiment, and three independent experiments were performed. Data are presented as the mean ± SD from the independent experiments.

### 4.15. Statistical Analysis

Statistical analyses were primarily performed using R software (version 4.6.1; R Foundation for Statistical Computing, Vienna, Austria). GraphPad Prism (version 9.5.0; GraphPad Software, Boston, MA, USA) was used for statistical analysis and visualization of cell-based experiments. Comparisons between two independent experimental groups were performed using unpaired two-tailed Student’s *t*-tests assuming equal variances. For time-course cell-viability experiments, control and ATPSCKMT-knockdown groups were compared separately at each time point using unpaired Student’s *t*-tests, and multiple comparisons were corrected using the two-stage linear step-up procedure of Benjamini, Krieger, and Yekutieli. Pan-cancer tumor-versus-normal expression differences were evaluated using the Wilcoxon rank-sum test. Associations between continuous gene-expression or pathway variables were assessed using Spearman rank correlation. Survival associations were evaluated using Cox proportional hazards regression and Kaplan–Meier analysis with the log-rank test. Meta-analyses of cohort-specific correlation coefficients were performed using the metafor R package with a random-effects restricted maximum-likelihood (REML) model. *p*-values from high-dimensional analyses were adjusted using the Benjamini–Hochberg method where indicated. Unless otherwise stated, a two-sided *p* < 0.05 was considered statistically significant. For analyses involving multiple-testing correction, an FDR < 0.05 was considered statistically significant. Cell-based experimental data are presented as the mean ± standard deviation (SD). For the copy-number-adjusted ATPSCKMT–CCT5 analysis, partial Spearman correlation was implemented by rank transformation and residualization against the ranked shared copy-number state, with 95% confidence intervals calculated using Fisher’s z transformation. Candidate-gene detection frequencies in single cells were compared using Fisher’s exact test for pooled cell-level analyses and paired Wilcoxon signed-rank tests for patient-level analyses. Sequencing-depth metrics were compared using the Wilcoxon rank-sum test. ChatGPT (GPT-6; OpenAI) was used to assist with the drafting and refinement of R code used for data analysis. All code, analytical procedures, and outputs were independently reviewed, executed, and validated by the authors, who retained full responsibility for all analytical decisions and interpretations.

## 5. Conclusions

In conclusion, pan-cancer analysis of METTL/7BS KMT genes revealed recurrent genomic alterations, expression dysregulation, and associations with patient outcomes. Integration of expression, prognosis, and RNAi dependency data prioritized LIHC for further investigation of ATPSCKMT, with this ranking remaining stable across alternative three-domain weighting schemes. ATPSCKMT knockdown reduced CCK-8 signals and EdU incorporation in two HCC cell lines, and its expression was associated with proliferation-related Hallmark programs across HCC cohorts. Together, these findings support a potential role for ATPSCKMT in proliferation-associated phenotypes, although the underlying mitochondrial mechanism was not directly examined. CCT5 emerged as a reproducible ATPSCKMT co-expression candidate across independent HCC cohorts, with the correlation persisting after adjustment for shared local copy-number variation. Its established involvement in CCT/TRiC-dependent protein folding provides biological context, but the present data do not establish a direct functional relationship with ATPSCKMT. Further mechanistic studies are warranted to clarify these findings.

## Figures and Tables

**Table 1:** Classification and molecular characteristics of METTL family and seven-β-strand lysine methyltransferase genes included in this study. Genes were classified into three groups: METTL family genes also classified as seven-β-strand lysine methyltransferases (METTL family + 7BS KMT), METTL family genes not classified as 7BS lysine methyltransferases (METTL family only), and non-METTL genes classified as 7BS lysine methyltransferases (7BS KMT only). Current gene symbols were used throughout the table, with representative former or alternative symbols provided where applicable. Chromosomal locations are presented as cytogenetic bands based on the human GRCh38 assembly. UniProtKB accessions, amino-acid sequence lengths, and molecular weights correspond to canonical human protein entries. Molecular weights are expressed in kilodaltons (kDa).

Gene Symbol	Alternative Symbol	Chromosomal Location	UniProtKB Accession	Sequence Length (aa)	MW (kDa)
**METTL family + 7BS KMT (n = 9)**					
CSKMT	METTL12	11q12.3	A8MUP2	240	25.9
EEF1AKMT2	METTL10	10q26.13	Q5JPI9	236	25.9
EEF1AKMT3	METTL21B	12q14.1	Q96AZ1	226	24.9
ETFBKMT	METTL20	12p11.21	Q8IXQ9	262	29.5
METTL13	—	1q24.3	Q8N6R0	699	78.8
METTL21A	—	2q33.3	Q8WXB1	218	24.6
METTL21C	—	13q33.1	Q5VZV1	264	29.6
METTL22	—	16p13.2	Q9BUU2	404	44.5
VCPKMT	METTL21D	14q21.3	Q9H867	229	25.8
**METTL family only (n = 24)**					
METTL1	—	12q14.1	Q9UBP6	276	31.5
METTL2A	—	17q23.2	Q96IZ6	378	43.5
METTL2B	—	7q32.1	Q6P1Q9	378	43.4
METTL3	—	14q11.2	Q86U44	580	64.5
METTL4	—	18p11.32	Q8N3J2	472	54.0
METTL5	—	2q31.1	Q9NRN9	209	23.7
METTL6	—	3p25.1	Q8TCB7	284	33.3
METTL8	—	2q31.1	Q9H825	291	33.4
METTL9	—	16p12.2	Q9H1A3	318	36.5
METTL14	—	4q26	Q9HCE5	456	52.2
METTL15	—	11p14.1	A6NJ78	407	46.1
METTL16	—	17p13.3	Q86W50	562	63.6
METTL17	METT11D1	14q11.2	Q9H7H0	456	50.7
METTL18	—	1q24.2	O95568	372	42.1
METTL23	—	17q25.1	Q86XA0	190	21.5
METTL24	C6orf186	6q21	Q5JXM2	366	41.3
METTL25	C12orf26	12q21.31	Q8N6Q8	603	68.2
METTL26	C16orf13	16p13.3	Q96S19	204	22.6
METTL27	WBSCR27	7q11.23	Q8N6F8	245	26.5
NTMT1	METTL11A	9q34.11	Q9BV86	223	25.4
NTMT2	METTL11B	1q24.2	Q5VVY1	283	32.4
TMT1A	METTL7A	12q13.12	Q9H8H3	244	28.3
TMT1B	METTL7B	12q13.2	Q6UX53	244	27.8
TRMT44	METTL19	4p16.1	Q8IYL2	757	84.6
**7BS KMT only (n = 5)**					
ANTKMT	FAM173A	16p13.3	Q9BQD7	235	25.1
ATPSCKMT	FAM173B	5p15.2	Q6P4H8	233	26.1
EEF1AKMT1	N6AMT2	13q12.11	Q8WVE0	214	24.5
EEF1AKMT4	—	3q27.1	P0DPD7	255	28.3
EEF2KMT	FAM86A	16p13.3	Q96G04	330	36.9

## Data Availability

The publicly available datasets analyzed in this study were obtained from the Genomic Data Commons (GDC), cBioPortal for Cancer Genomics, the Cancer Dependency Map (DepMap), the Hepatocellular Carcinoma Database (HCCDB), and the Gene Expression Omnibus (GEO; accession GSE149614). Protein-interaction information was obtained from STRING and BioGRID. Data generated from the cell-based experiments in this study are available from the corresponding author upon reasonable request.

## Supplementary Materials

The following supporting information can be downloaded at https://www.mdpi.com/article/10.3390/molecules31213887/s1: Table S1: siRNA sequences used for ATPSCKMT knockdown; Table S2: Primer sequences used for quantitative real-time PCR; Figure S1: Somatic mutation distribution and overall survival according to mutation status of METTL3 and METTL4; Figure S2: Pan-cancer CRISPR dependency landscape of METTL and 7BS lysine methyltransferase genes; Figure S3: Complete-case and weighting sensitivity analyses of ATPSCKMT prioritization in LIHC; Figure S4: Expression patterns of METTL and 7BS lysine methyltransferase genes across HCC transcriptomic cohorts; Figure S5: Copy-number-adjusted association between ATPSCKMT and CCT5 expression in TCGA-LIHC; Figure S6: Patient-level distribution of ATPSCKMT and candidate gene detection in malignant hepatocytes.

## Abbreviations

The following abbreviations are used in this manuscript:

7BS	Seven-β-strand
CCK-8	Cell Counting Kit-8
CCT	Chaperonin-containing TCP-1
CI	Confidence interval
DAPI	4′,6-Diamidino-2-phenylindole
DMEM	Dulbecco’s modified Eagle’s medium
EdU	5-Ethynyl-2′-deoxyuridine
FDR	False discovery rate
GDC	Genomic Data Commons
GEO	Gene Expression Omnibus
HCC	Hepatocellular carcinoma
HCCDB	Hepatocellular Carcinoma Database
HR	Hazard ratio
KMT	Lysine methyltransferase
LIHC	Liver hepatocellular carcinoma
METTL	Methyltransferase-like
MSigDB	Molecular Signatures Database
OS	Overall survival
PFI	Progression-free interval
qPCR	Quantitative real-time polymerase chain reaction
REML	Restricted maximum likelihood
RNAi	RNA interference
SAM	S-Adenosyl-L-methionine
SD	Standard deviation
siRNA	Small interfering RNA
ssGSEA	Single-sample gene set enrichment analysis
TCGA	The Cancer Genome Atlas
TCGA-CDR	TCGA Pan-Cancer Clinical Data Resource
TPM	Transcripts per million
TRiC	TCP-1 ring complex
UMAP	Uniform Manifold Approximation and Projection
