# Supplementary material for: Integrated Pan-Cancer Analysis and Functional Characterization of ATPSCKMT in Hepatocellular Carcinoma
Source: Molecules. 2026 Oct 28;31(21):3887. doi: 10.3390/molecules31213887 (PMC13765333; doi:10.3390/molecules31213887)
Supplement: Supplementary file 1 [file molecules-31-03887-s001.zip › molecules-4602391-supplementary.pdf]

Supplementary Materials

Table S1. siRNA sequences used for ATPSCKMT knockdown. Two independent siRNAs targeting ATPSCKMT were used as a pool. Sense (S) and antisense (AS) strands are shown in the 5′→3′ orientation. A non-targeting siRNA was used as the control.

Gene Name	siRNA	Strand	Sequence (5′–3′)
ATPSCKMT	siRNA #1	Antisense (AS)	GAGAAGAGACUUGACUGAU
		Sense (S)	AUCAGUCAAGUCUCUUCUC
	siRNA #3	Antisense (AS)	GAGACUUGACUGAUUUAGA
		Sense (S)	UCUAAAUCAGUCAAGUCUC

Table S2. Primer sequences used for quantitative real-time PCR. F, forward primer; R, reverse primer. Sequences are shown in the 5′→3′ orientation.

Gene Name	Primer	Sequence (5′–3′)
ATPSCKMT	Forward:	GCCGTTTGTACCTGCAACTACG
	Reverse:	CCTTTCTTCGCAGCCGCTATGA
GAPDH	Forward:	GCACCGTCAAGGCTGAGAAC
	Reverse:	TGGTGAAGACGCCAGTGGA

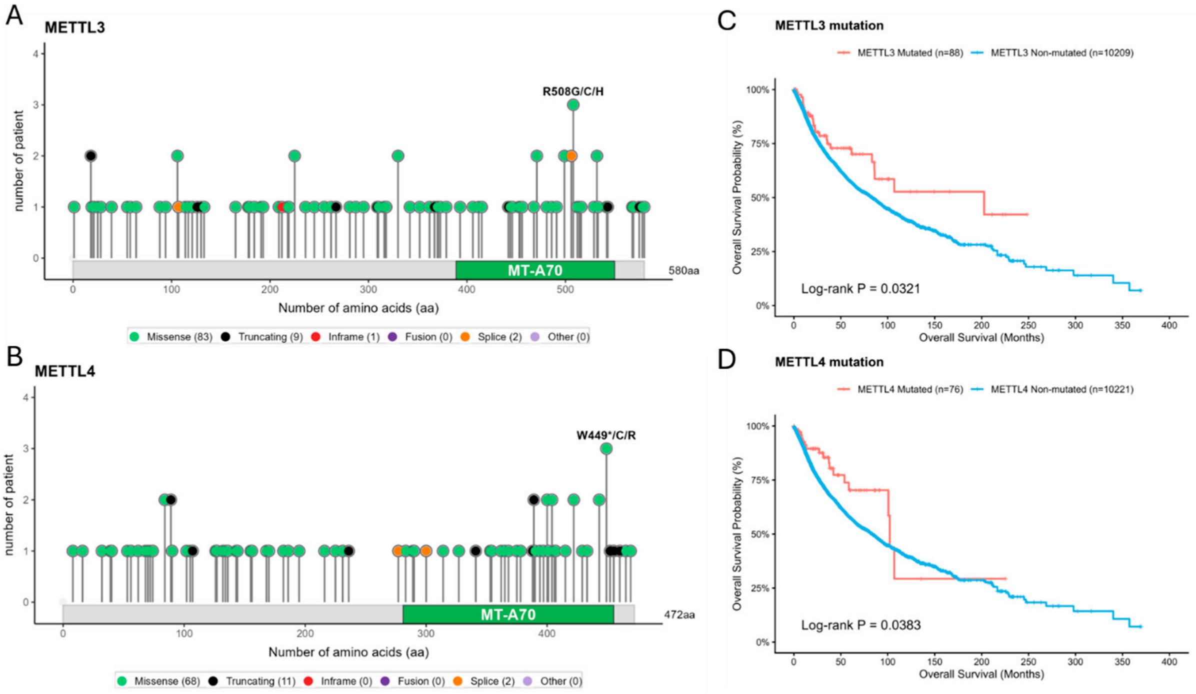

Figure S1. Somatic mutation distribution and overall survival according to mutation status of METTL3 and METTL4. (A,B) Distribution of somatic mutations across the protein sequences of METTL3 (A) and METTL4 (B), the two genes with the highest mutation frequencies in the pan-cancer analysis. Mutation classes, recurrently altered residues, and annotated protein domains are indicated where applicable. (C,D) Kaplan–Meier analyses of overall survival according to mutation status for METTL3 (C) and METTL4 (D). Survival distributions were compared using the log-rank test, and the corresponding P values are indicated in each panel.

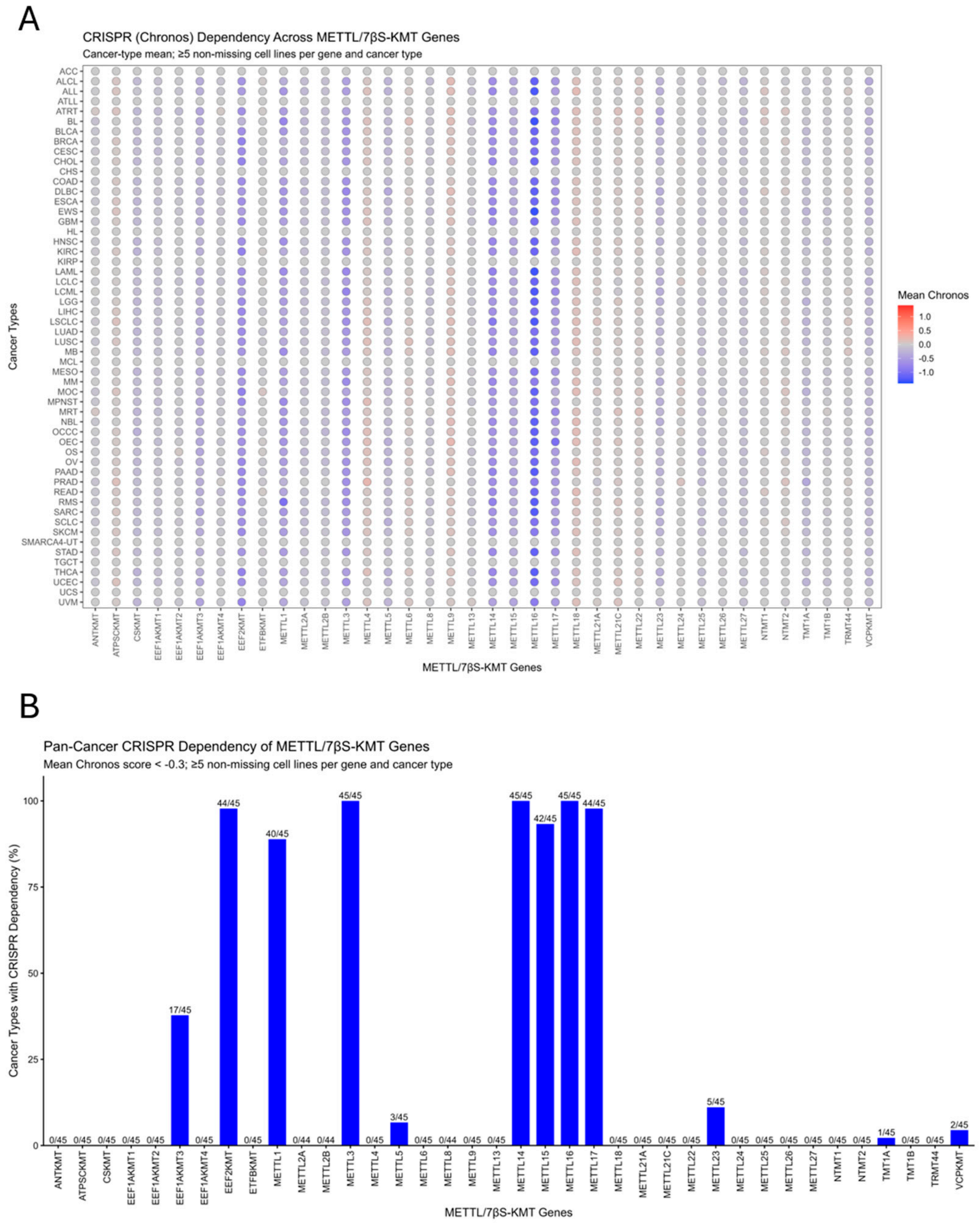

Figure S2. Pan-cancer CRISPR dependency landscape of METTL and 7BS lysine methyltransferase genes. (A) Pan-cancer CRISPR dependency profiles of METTL/7BS KMT genes based on Chronos gene-effect scores from DepMap. Mean Chronos scores were calculated for each gene within each cancer type using only gene–cancer type combinations with at least five non-missing cell lines. More negative Chronos scores indicate greater dependency. (B) Proportion of evaluable cancer types meeting the descriptive dependency criterion for each METTL/7BS KMT gene. Dependency was defined as a mean Chronos score < −0.3. Numbers above the bars indicate the number of cancer types meeting the criterion relative to the total number of evaluable cancer types for each gene. The numbers of evaluable cancer-type categories differ between the Chronos and DEMETER2 datasets and the corresponding proportions are therefore not directly comparable.

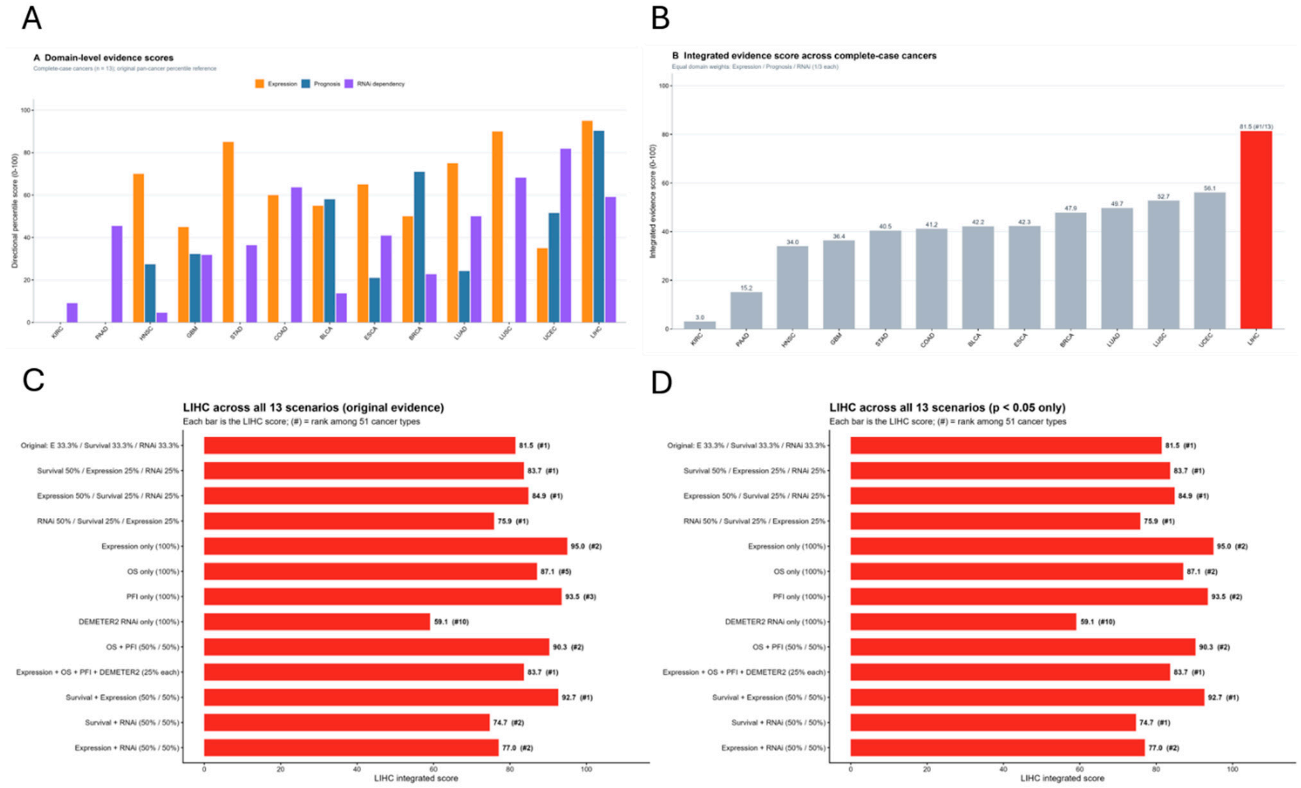

Figure S3. Complete-case and weighting sensitivity analyses of ATPSCKMT prioritization in LIHC. (A) Domain-specific evidence scores for 13 cancer types with complete expression, prognosis, and RNAi dependency data. Complete-case cancer types were selected from the original 51 cancer types, and directional percentile scores were recalculated exclusively within the 13 eligible cancer types. Orange, blue, and purple bars represent expression, prognosis, and RNAi dependency scores, respectively. (B) Integrated evidence scores for the 13 complete-case cancer types, calculated using equal weighting of expression, prognosis, and RNAi dependency. LIHC is highlighted in red, whereas the remaining cancer types are shown in gray. LIHC achieved the highest integrated score (91.7), followed by UCEC (62.5) and LUSC (61.1). (C) LIHC integrated scores and rankings across 13 scoring scenarios evaluated among the original 51 cancer types. These scenarios included alternative domain weighting, individual indicators, pairwise combinations, and equal weighting of four individual indicators. (D) Corresponding LIHC scores and rankings after applying a nominal P-value threshold of 0.05 to expression, OS, and PFI evidence while retaining the original RNAi scores. In panels A and B, missing evidence was excluded through complete-case selection, and percentile scores were recalculated within the eligible subset. In panels C and D, the original pan-cancer scoring framework was retained, with unavailable evidence contributing zero to the integrated score. Numbers in parentheses in panels C and D indicate LIHC rankings among 51 cancer types.

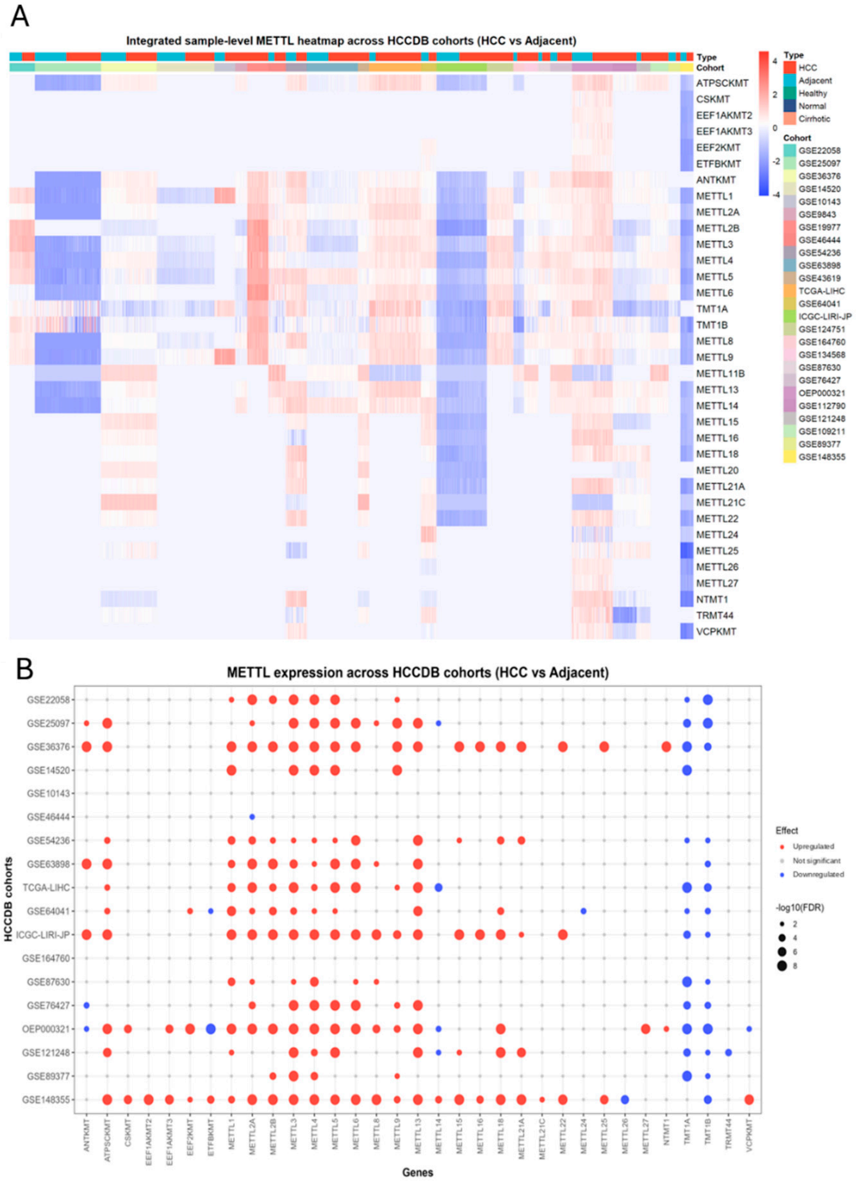

Figure S4. Expression patterns of METTL and 7BS lysine methyltransferase genes across HCC transcriptomic cohorts.

(A) Integrated sample-level heatmap showing expression patterns of METTL/7BS KMT genes across HCC transcriptomic datasets. Samples are annotated according to tissue type and cohort of origin. Expression values were standardized within the corresponding analysis framework prior to visualization. (B) Cohort-specific differential expression of METTL/7BS KMT genes between HCC and adjacent non-tumor tissues. Dot color indicates the direction of differential expression, and dot size represents statistical significance based on the FDR-adjusted P value. Non-significant comparisons are indicated separately.

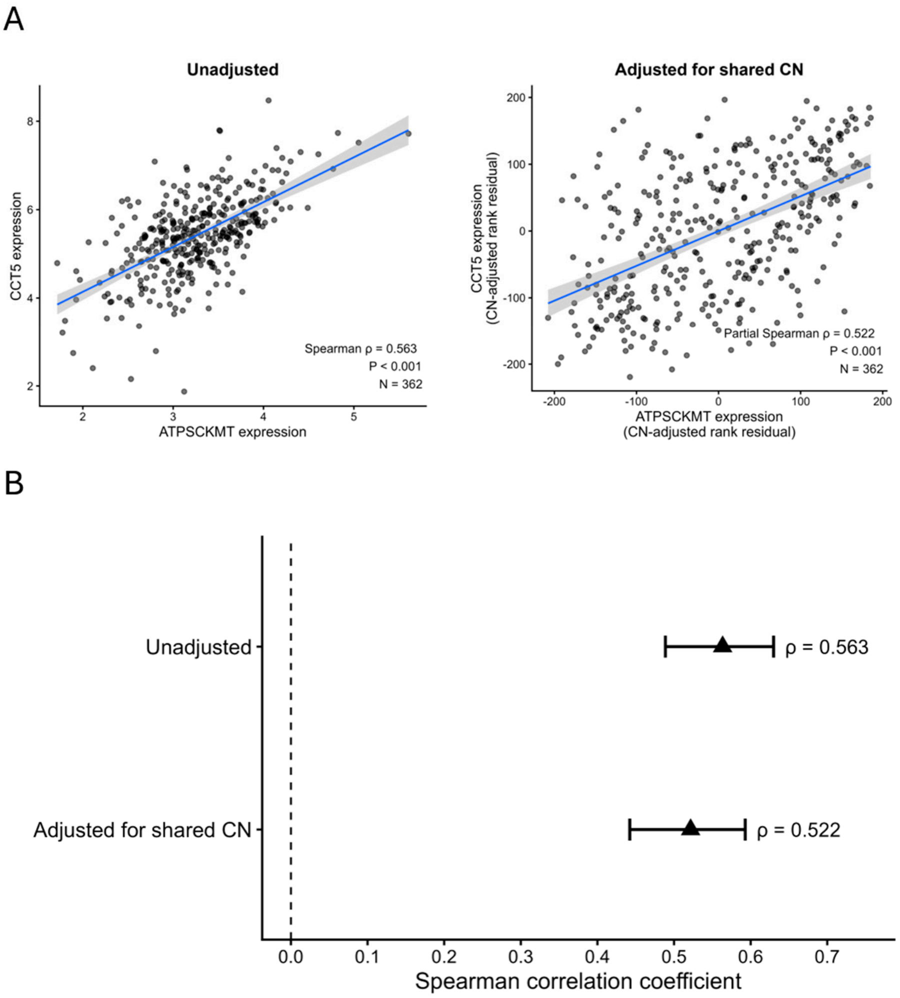

Figure S5. Copy-number-adjusted association between ATPSCKMT and CCT5 expression in TCGA-LIHC. (A) Association between ATPSCKMT and CCT5 expression in TCGA-LIHC tumors before and after adjustment for local copy-number status. The left panel shows the unadjusted relationship between ATPSCKMT and CCT5 expression (Spearman ϱ = 0.563, P < 0.001, N = 362). Because ATPSCKMT and CCT5 showed identical ASCAT3 gene-level copy-number states across the matched tumors, a single shared local copy-number variable was used for adjustment. For the adjusted analysis, expression and copy-number values were rank-transformed, the effect of the shared copy-number state was removed by linear regression, and the residualized expression ranks were used to calculate the partial Spearman correlation (ϱ = 0.522, P < 0.001, N = 362). Lines indicate fitted linear trends with 95% confidence bands. (B) Comparison of the unadjusted and copy-number-adjusted correlation coefficients between ATPSCKMT and CCT5 expression. Triangles indicate the correlation coefficients, and horizontal bars represent 95% confidence intervals.

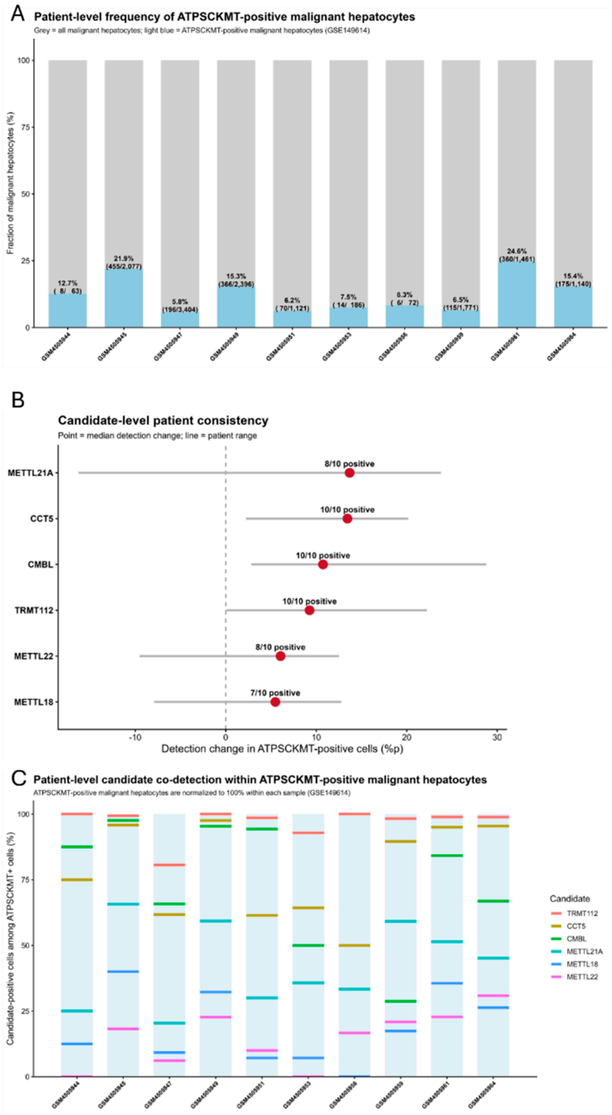

Figure S6. Patient-level distribution of ATPSCKMT and candidate gene detection in malignant hepatocytes. (A) Patient-level frequency of ATPSCKMT-positive malignant hepatocytes. For each patient, the total malignant-hepatocyte population is normalized to 100%, and the light-blue portion indicates the fraction with detectable ATPSCKMT expression. Percentages and cell counts are indicated for each patient. (B) Candidate-level summary of patient-specific differences in gene detection between ATPSCKMT-positive and ATPSCKMT-negative malignant hepatocytes. Points indicate the median detection difference across patients, and horizontal lines indicate the observed patient range. Labels indicate the number of patients with a positive detection difference. (C) Patient-level detection frequencies of the indicated candidate genes among ATPSCKMT-positive malignant hepatocytes. For each patient, ATPSCKMT-positive malignant hepatocytes are normalized to 100%, and colored horizontal markers indicate the percentage of cells in which each candidate gene was detected.
